# Inhibition of Non-Small Cell Lung Cancer Cells by Oxy210, an Oxysterol-Derivative that Antagonizes TGFβ and Hedgehog Signaling

**DOI:** 10.3390/cells8101297

**Published:** 2019-10-22

**Authors:** Frank Stappenbeck, Feng Wang, Liu-Ya Tang, Ying E. Zhang, Farhad Parhami

**Affiliations:** 1 MAX BioPharma, Inc., 2870 Colorado Avenue, Santa Monica, CA 90404, USA; fstappenbeck@maxbiopharma.com (F.S.); fwang@maxbiopharma.com (F.W.); 2 Laboratory of Cellular & Molecular Biology, Center for Cancer Research, National Cancer Institute, 37 Convent Drive, 37/2056B Bethesda, MD 20892-4256, USA; tangl2@mail.nih.gov (L.-Y.T.); zhangyin@mail.nih.gov (Y.E.Z.)

**Keywords:** TGFβ signaling, hedgehog signaling, oxysterols, metastasis, drug resistance

## Abstract

Non-Small Cell Lung Cancer (NSCLC) is a common malignancy and leading cause of death by cancer. Metastasis and drug resistance are serious clinical problems encountered in NSCLC therapy. Aberrant activation of the Transforming Growth Factor beta (TGFβ) and Hedgehog (Hh) signal transduction cascades often associate with poor prognosis and aggressive disease progression in NSCLC, as these signals can drive cell proliferation, angiogenesis, metastasis, immune evasion and emergence of drug resistance. Therefore, simultaneous inhibition of TGFβ and Hh signaling, by a single agent, or in combination with other drugs, could yield therapeutic benefits in NSCLC and other cancers. In the current study, we report on the biological and pharmacological evaluation of Oxy210, an oxysterol-based dual inhibitor of TGFβ and Hh signaling. In NSCLC cells, Oxy210 inhibits proliferation, epithelial-mesenchymal transition (EMT) and invasive activity. Combining Oxy210 with Carboplatin (CP) increases the anti-proliferative response to CP and inhibits TGFβ-induced resistance to CP in A549 NSCLC cells. In addition, Oxy210 displays encouraging drug-like properties, including chemical scalability, metabolic stability and oral bioavailability in mice. Unlike other known inhibitors, Oxy210 antagonizes TGFβ and Hh signaling independently of TGFβ receptor kinase inhibition and downstream of Smoothened, respectively.

## 1. Introduction

Non-Small Cell Lung Cancer (NSCLC), which accounts for 85% of all lung cancers, is a common malignancy and leading cause of death by cancer worldwide, implicated in nearly 20% of all cancer mortality [1,2]. Despite many available generic treatment options, such as chemotherapy, radiation therapy, surgery and decades of research and clinical experimentation with promising approaches and new cancer drugs, NSCLC continues to be associated with poor prognoses and a lower five-year survival rate than many other forms of cancer [2]. While the five-year survival rate for metastatic lung cancer stands at less than 5%, the outlook for cases diagnosed at an early stage is much improved. However, only a minority of NSCLC cases can be diagnosed and treated while the tumor is still localized within the lungs. Metastasis and drug resistance are known to be major causes of failure in NSCLC therapy. Metastases occur frequently in NSCLC, locally into lymph nodes and the thoracic wall as well as into distant tissues, such as the brain, bones and the adrenal glands [3]. New medicines are needed to not only inhibit tumor growth but also reduce invasive activity and metastasis and help overcome drug resistance in NSCLC patients.

Rather than a singular disease entity, NSCLC has been described, as a spectrum of multiple pathologies with unique histological and molecular signatures, characterized by a progressive loss of tumor suppressor function and the emergence of oncogenic mutations that cause aberrant cellular signaling and drive disease progression [4]. In NSCLC, as well as other cancers, oncogenic driver mutations can occur in key signal transduction molecules, often receptor tyrosine kinases, associated with cellular signaling pathways relevant to NSCLC tumor cell survival and proliferation. Prominent examples of mutated signaling proteins in NSCLC include: the epidermal growth factor receptor (EGFR), the Kirsten rat sarcoma viral oncogene homolog GTPase (KRAS), BRAF, a member of the rapidly accelerated fibrosarcoma (RAF) family and the fusion oncogene echinoderm microtubule associated protein-like 4-anaplastic lymphoma receptor tyrosine kinase (EML4-ALK). The clinical development of kinase inhibitors as targeted therapies for oncogenic driver mutations has been quite successful for some mutations (e.g., EGFR and BRAF), but less so with others (e.g., KRAS), and there are no current therapies targeting tumor suppressor loss [5]. Kinase inhibitors, such as Erlotinib, Gefitinib, Dabrafenib, Trametinib or Crizotinib, often given in combination with other drugs, can temporarily reduce tumor burden, extend overall survival and improve the quality of life in select NSCLC patients; however, these treatments are rarely curative due to near universal development of drug resistance and tumor relapse, after initially promising responses [6]. A significant fraction of NSCLC tumors does not present a mutational profile that can currently be targeted. For these patients, platin based drugs, such as Carboplatin (CP) or Cisplatin, are used as a standard of care, often given in combination with Pemetrexed, Gemcitabine or other cytotoxic drugs [7]. Unfortunately, due to the refractory nature of NSCLC and development of drug resistance, the resulting survival benefits of platin-based therapies are often only modest and transient. More recently, immunotherapy drugs, such as the PD-1 immune checkpoint signaling inhibitor pembrolizumab (Keytruda), have been introduced as first-line therapy in some NSCLC patients, either alone or in combination with other drugs, but are used more often in patients with recurrent NSCLC [8].

Besides driver mutations and a loss of tumor suppressor function, aberrant reactivation of embryonic cellular signaling in tumor cells contributes significantly to metastasis and drug resistance in NSCLC and other cancers [9]. The gene expression repertoire of embryonic development can profoundly affect both cancerous and non-cancerous cells, including lung fibroblasts, cancer associated fibroblasts and immune cells. Among several developmental signaling pathways broadly implicated in cancer, such as Fibroblast Growth Factor (FGF), Wnt and Notch signaling, the Transforming Growth Factor Beta (TGFβ) and Hedgehog (Hh) signaling pathways are notable for their partially overlapping effects on the tumor microenvironment, cell proliferation, angiogenesis, metastasis, immune evasion and emergence of drug resistance in NSCLC [10]. The TGFβ and Hh signaling pathways are both necessary for proper lung organogenesis, postnatal lung homeostasis and tissue remodeling after lung injury [11,12]. Both pathways are well known for their cross talk with each other and other pathways through non-canonical signaling [13,14]. Mechanistically, TGFβ and Hh signaling are reported to intersect at multiple levels, such as, for example, the transcription factor Gli2 which is a TGFβ target gene as well as a regulator of Hh signaling and at the level of SMAD4 and Gli1 [15,16]. In the proper context, therefore, simultaneous inhibition of both TGFβ and Hh signaling by a single agent, used alone or in combination with other drugs (i.e., cytotoxic or targeted therapies), could yield significant therapeutic benefits in NSCLC across a wide range of mutational profiles [17,18] by targeting both tumor cells, as well as the tumor microenvironment [3,19]. Furthermore, inhibition of TGFβ and/or Hh signaling, may also be beneficial when combined with immunotherapy drugs, such as PD-1/PDL-1 inhibitors, which is a topic of several ongoing clinical investigations [20]. 

**Figure 1:**
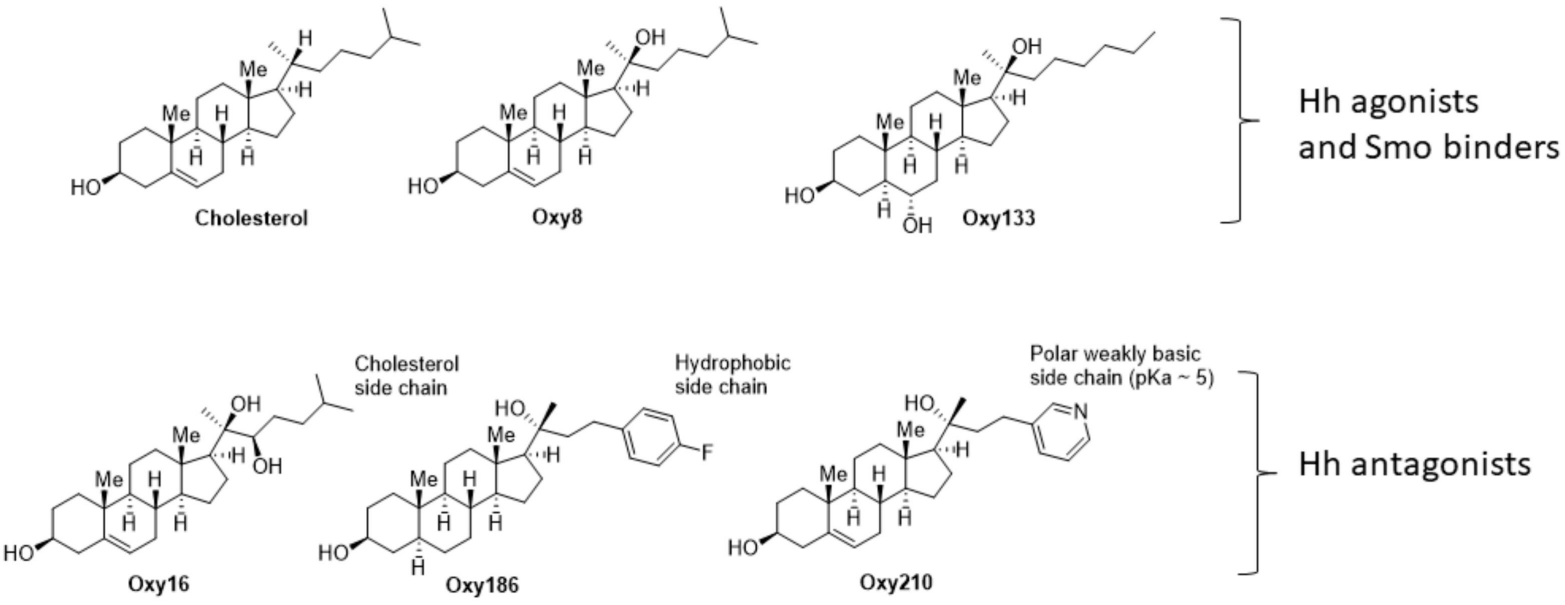
The molecular structures of Cholesterol, Oxy8, Oxy133, Oxy16, Oxy186 and Oxy210.

Oxysterols, defined as oxidized derivatives of cholesterol, can be activators or inhibitors of cellular signaling [21]. We previously reported on the ability of Oxy16 (20α,22(*R*)-dihydroxycholesterol, Figure 1), a naturally occurring oxysterol, to block Hh signaling downstream of the Smoothened (Smo) receptor, a plasma membrane-associated signal transducer of Hh signaling that mediates ligand activated Hh signaling [22]. In subsequent studies, through analysis of structure activity relationships (SAR) between analogues of Oxy16, we were able to identify more potent semisynthetic oxysterol derivatives, such as Oxy186, that inhibit Hh signaling and the proliferation of NSCLC and pancreatic tumor cells in vitro [23]. More recently, we have identified structural analogues of Oxy186 that in addition to their Hh pathway inhibitory properties also block TGFβ signaling. These oxysterol analogues, such as our lead compound Oxy210 reported here, present a unique opportunity to investigate the role of Hh and TGFβ signaling in the pathogenesis of human malignancies, including NSCLC.

In the current study, we report on the biological and pharmacological evaluation of Oxy210, as a dual inhibitor of TGFβ and Hh signaling with encouraging drug-like properties, including chemical scalability, metabolic stability, oral bioavailability in mice and initial drug safety paneling. Based on in vitro activities of Oxy210 in NSCLC tumor cells, blocking their proliferation, epithelial-mesenchymal transition (EMT), invasive activity, inhibition of their TGF-β induced drug resistance and the pharmacological profile presented here, Oxy210 may be a suitable candidate for future studies directed toward therapeutic development in NSCLC. 

## 2. Materials and Methods

### 2.1. Synthesis and Molecular Characterization of Oxy210 and Oxy16

Materials were obtained from commercial suppliers and were used without further purification. Air or moisture sensitive reactions were conducted under an argon atmosphere using oven-dried glassware and standard syringe/septa techniques. The reactions were monitored on silica gel TLC plates under UV light of 254 nm (UVP, Upland, CA, USA) followed by visualization with Hanessian’s staining solution. Chromatographic purifications were performed using a Teledyne ISCO CombiFlash Rf automated chromatography system (Lincoln, NE, USA). NMR (Bruker Corporation, Billerica, MA, USA) spectra were measured in CDCl3. The data are reported as follows in ppm from an internal standard (TMS, 0.0 ppm): chemical shift (multiplicity, integration, coupling constant in Hz).

### 2.2. Synthesis and Crystallographic Analysis of Oxy210

**Scheme 1:**
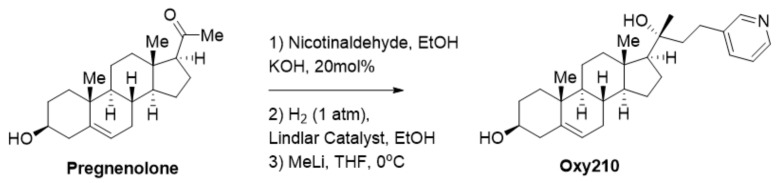
Synthesis of Oxy210.

**Figure 2:**
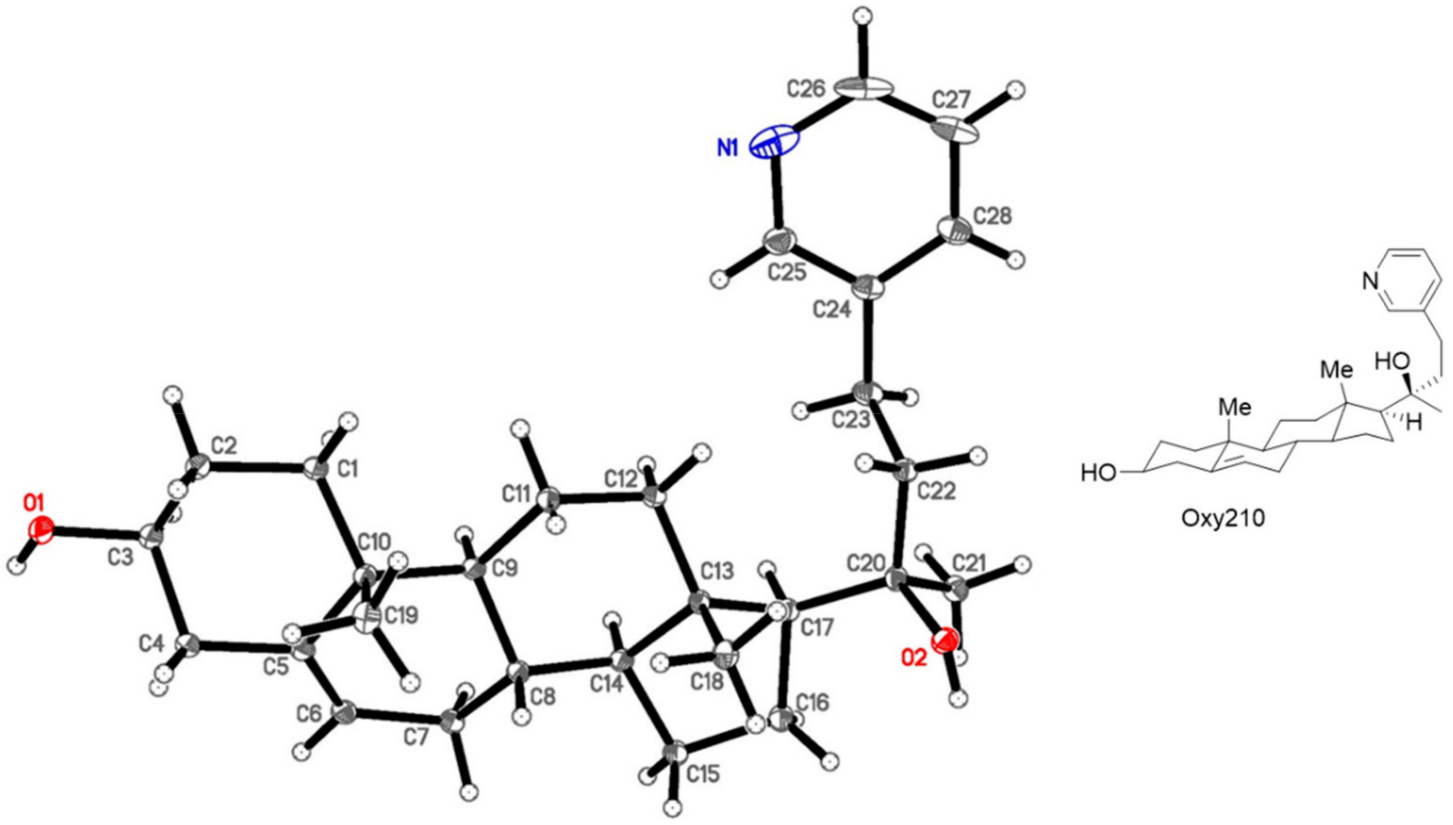
Ortep representation of Oxy210 in the solid state.

(*3S,8S,9S,10R,13S,14S,17S*)-17-((*R*)-2-hydroxy-4-(pyridin-3-yl)butan-2-yl)-10,13-dimethyl-2,3,4,7,8,9,10,11,12,13,14,15,16,17-tetradecahydro-1H-cyclopenta[a]phenanthren-3-ol (Oxy210): Oxy210 was prepared in three synthetic steps depicted in Scheme 1. Briefly, pregnenolone was condensed with nicotinaldehyde to the enone which was reduced to a ketone via hydrogenation using Lindlar’s catalyst. The ketone was reacted with methyl lithium to afford the 20(*R*)-tertiary alcohol, Oxy210. The crude product was purified by chromatography on silica. ^1^H NMR (400 MHz, CDCl_3_) δ 8.45 (1H, d, *J* = 1Hz), 8.42 (1H, dd, *J* = 5, 2 Hz), 7.53–7.48 (1H, m), 7.23–7.18 (1H, m), 5.35–5.31 (1H, m), 3.56–3.45 (1H, m), 2.79–2.63 (2H, m), 2.33–2.17 (2H, m), 2.05 (1H, m), 2.01–1.26 (16 H, m), 1.23 (3H, s), 1.18–0.89 (3H, m), 0.98 (3H, s), 0.87 (3H, s); ^13^C NMR (100 MHz, CDCl3) δ 149.7, 147.1, 140.8, 138.1, 135.8, 128.6, 123.4, 121.4, 75.5, 71.6, 58.7, 56.9, 50.0, 44.1, 42.9, 42.3, 40.3, 37.2, 36.5, 31.7, 31.6, 31.3, 27.5, 26.7, 23.7, 23.2, 20.9, 19.3, 13.7. MS (ESI + ve): [M + H] = 424.31 conforms to structural formula C_28_H_41_NO_2_, MW = 423.31. A 5 mg portion of Oxy210 was dissolved in MeOH (0.5 mL) and crystallization was induced by slow evaporation of the solvent. Single crystal X-ray diffraction data were collected at 100K on a diffractometer with Bruker Apex-II CCD detector and a Cu-micro focus source. Crystal data: Orthorhombic, a = 7.2736(2) Å, b = 13.3504(4) Å, c = 26.2632(8) Å, α = 90° β = 90°, γ = 90°, Vol. = 2550.30(13) Å3, Space group = P212121. The final anisotropic full matrix least-squares refinement on F2 converged at R1 = 0.0319, wR2 = 0.088, GOF = 1.01. Analysis of the structure indicates that observed conformational details are intra-molecularly controlled and that the influence of crystal packing forces on the sterol conformation is negligible. Figure 2: Ortep representation of Oxy210 in the solid state.

### 2.3. Synthesis and Crystallographic Analysis of Oxy16

**Figure 3:**
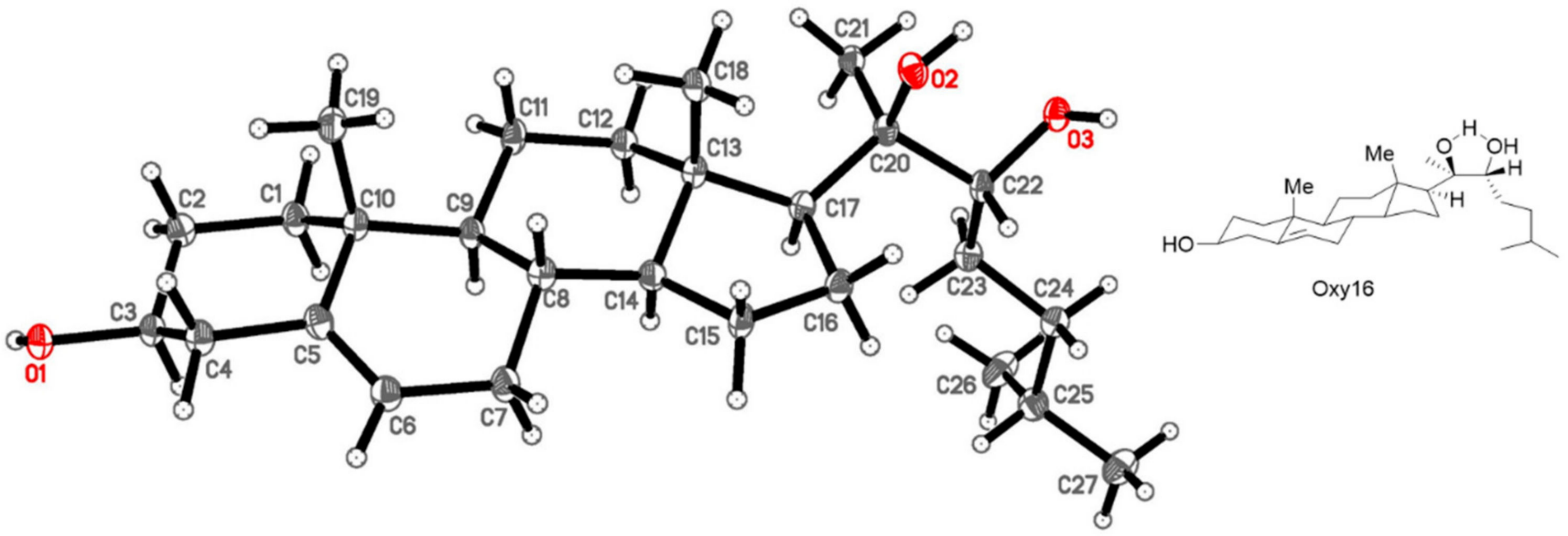
Ortep representation of Oxy16 in the solid state.

(2*R*,3*R*)-2-((3*S*,8*S*,9*S*,10*R*,13*S*,14*S*,17*S*)-3-hydroxy-10,13-dimethyl-2,3,4,7,8,9,10,11,12,13,14,15,16,17-tetradecahydro-1H-cyclopenta[a]phenanthren-17-yl)-6-methylheptane-2,3-diol (Oxy16): Oxy16 (20α,22(*R*)-dihydroxycholesterol) was prepared in four steps as previously described by Watanabe et al. [24] ^1^H NMR (CDCl_3_, 300 MHZ) δ: 5.36 (1H, m), 3.51 (1H, m), 3,41 (1H, m), 2.41 − 1.5 (15H, m), 1.22–0.95 (5H, m), 1.21 (3H, s), 1.01 (3H, s), 0.91 (3H, d, *J* = 6 Hz), 0.90 (3H, d, *J* = 6 Hz), 0.89 (3H, s). ^13^C NMR (CDCl_3_, 75 MHZ) δ: 140.69, 121.44, 76.30, 71.63, 56.6, 54.65, 49.96, 43.10, 42.16, 40.10, 37.15, 36.40, 36.24, 31.66, 31.52, 31.19, 29.07, 27.98, 23.84, 22.84, 22.26, 21.84, 20.85, 20.29, 19.29, 13.47. MS (ESI): mass calcd. for C_27_H_42_O, 383.3314; m/z found, 383.3326 [M − 2 H_2_O]^+^. A 5 mg portion of Oxy16 was dissolved in EtOAc (0.5 mL) and crystallization was induced by slow evaporation of the solvent. X-ray diffraction data were collected at 100K on a diffractometer with Bruker Apex-II CCD detector and a Cu-micro focus source. Crystal data: Monoclinic, a = 6.2273(2) Å, b = 27.949(1) Å, c = 17.2896(6) Å, β = 91.788(2), Vol. = 3007.7(2) Å3, Space group = P21. The structure was refined as a two-component twin and the final anisotropic full matrix least-squares refinement on F2 converged at R1 = 0.054, wR2 = 0.153, GOF = 1.08. Analysis of the structure indicates that observed conformational details are intra-molecularly controlled and that the influence of crystal packing forces on the sterol conformation is negligible. Figure 3: Ortep representation of Oxy16 in the solid state.

### 2.4. Cell Culture and Reagents

NIH3T3-E1 fibroblasts were obtained from ATCC (Manassas, VA, USA) and cultured as previously described [25,26]. The human lung cancer cell lines A549 and H2030 were obtained from ATCC and cultured in RPMI-1640 medium containing 10% FBS and antibiotics as previously described [23]. CAPAN-1 human pancreatic adenocarcinoma cell line was purchased from ATCC and cultured in Dulbecco’s Modified Eagle Medium (DMEM) containing 10% FBS and antibiotics as previously described [23]. Sufu-/- mouse embryonic fibroblasts were a gift from Dr. Matthew Scott of Stanford University. TGFβRI-deficient mink lung epithelial cells, R1BL17, were maintained in MEM containing 10% FBS and non-essential amino acids as described [27]. Gant61, and SB431542 were obtained from Cayman Chemical Company (Ann Arbor, MI, USA), HPI-1 was obtained from Abcam (Cambridge, UK), TGFβ1 was obtained from R&D Systems (Minneapolis, MN, USA). Carboplatin was purchased from Cayman Chemical Company (Ann Arbor, MI, USA).

### 2.5. CAPAN-1 Conditioned Medium

Growth medium in confluent 10 cm2 tissue culture plates of CAPAN-1 cells was replaced with 10 mL of fresh growth medium and cells incubated for a total of 7 days. Conditioned medium (CM) containing Shh and Ihh was collected and spun down to remove any detached cells and debris, aliquoted, and stored frozen at −80 °C (21).

### 2.6. Quantitative RT-PCR 

Total RNA was extracted with the RNeasy Plus Mini Kit from Qiagen (Hilden, Germany) according to the manufacturer’s instructions. One microgram of RNA was reverse-transcribed using an iScript Reverse Transcription Supermix from Bio-Rad Laboratories (Hercules, CA, USA) to make single-stranded cDNA. The cDNAs were then mixed with Qi SYBR Green Supermix (Bio-Rad) for quantitative RT-PCR assay using a Bio-Rad I-cycler IQ quantitative thermocycler. All PCR samples were prepared in triplicate wells in a 96-well plate. After 40 cycles of PCR, melt curves were examined in order to ensure primer specificity. Fold changes in gene expression were calculated using the ΔΔCt method. Primers used for mouse were as follows: Oaz1 (5′-CCACTGCTTCGCCAGAGAG-3′) and (5′-CCCGGACCCAGGTTACTA-3′), Gli1 (5′- GCTTGGATGAAGGACCTTGTG-3′ and 5′- GCT GATCCAGCCTAAGGTTCTC-3′), Ptch1 (5′-CCATCGGCGACAAGAACC-3′ and 5′-CCAGCACAGCAAAGAAATACC-3′), Thbs1 (5′-GGGGAGATAACGGTGTGTTTG-3′ and 5′-CGGGGATCAGGTTGGCATT-3′), Cryab (5′-GTTCTTCGGAGAGCACCTGTT-3′ and 5′-GAGAGTCCGGTGTCAATCCAG-3′), Egr2 (5′-GCCAAGGCCGTAGACAAAATC-3′ and 5′-CCACTCCGTTCATCTGGTCA-3′), CTGF (5′-GGGCCTCTTCTGCGATTTC-3′ and 5′-ATCCAGGCAAGTGCATTGGTA-3′).

Primers used for human were as follows: GAPDH (5′- CCTCAAGATCATCAGCAATGCCTCCT-3′ and 5′- GGTCATGAGTCCTTCCACGATACCAA-3′), SHH (5′- CGGAGCGAGGAAGGGAAAG-3′) and (5′- TTGGGGATAAACTGCTTGTAGGC-3′), GLI1 (5′- GAAGCCGAGCCGAGTATC-3′ and 5′-GGTGAGTAGACAGAGGTTGG-3′), GLI2 (5′-GCCCTCACCTCCATCAATGC-3′ and 5′-ACTCACTGCTCTGCTTGTTCTG-3′), Fn-EDA (5′- AGGAAGCCGAGGTTTTAACTG-3′ and 5′- AGGACGCTCATAAGTGTCACC-3′), E-Cadherin (5′- ATTTTTCCCTCGACACCCGAT-3′ and 5′- TCCCAGGCGTAGACCAAGA-3′), SNAIL (5′- TCGGAAGCCTAACTACAGCGA-3′ and 5′- AGATGAGCATTGGCAGCGAG-3′), N-Cadherin (5′- TGCGGTACAGTGTAACTGGG-3′ and 5′- GAAACCGGGCTATCTGCTCG-3′), Vimentin (5′- AGTCCACTGAGTACCGGAGAC-3′ and 5′- CATTTCACGCATCTGGCGTTC-3′), ZO-1 (5′- CAACATACAGTGACGCTTCACA-3′ and 5′- CACTATTGACGTTTCCCCACTC-3′), MMP2 (5′- CCCACTGCGGTTTTCTCGAAT-3′ and 5′- CAAAGGGGTATCCATCGCCAT-3′), MMP9 (5′-TTGACAGCGACAAGAAGTGG-3′ and 5′-GCCATTCACGTCGTCCTTAT).

### 2.7. Transient Transfection Assay

NIH3T3-E1 cells cultured in 24 well-plates at 90% confluence were transiently transfected with 0.1 μg of Smad response-element reporter (SBE-Luciferase) plasmid (Qiagen), or with 0.1 μg of Gli response-element reporter (pGL3b-8xGli-Luciferase) plasmid, and 10 ng of pSV40-Renilla-Luciferase plasmid (Promega, Madison, WI, USA) with or without co-transfection with 10ng of Gli1 overexpression vector, pSRα-Gli1 using a Lipofectamine LTX Plus transfection reagent, as previously described [23]. Sufu-/- cells cultured in 24 well-plates at 90% confluence were transiently transfected with 0.1μg of pGL3b-8xGli-Luciferase plasmid, and 10 ng of pSV40-Renilla-Luciferase plasmid. 6 hrs after transfection, cells were treated with test agents for 72 h. R1BL17 cells cultured in 24 well-plates were transfected with 150 ng of TGFβRI plasmid (WT or TD mutant, which is constitutively active) [28], 100 ng of Smad responsive luciferase reporter (CAGA12-Luc), and 20 ng of pTK-Renilla-Luciferase plasmid using Lipofectamine P3000 transfection reagent (Invitrogen, Waltham, MA, USA). Twenty-four hours after transfection, cells were treated with test agents for 20 h. Then the firefly and Renilla luciferase activities were measured using a dual luciferase kit (Promega) and a GloMax-96 Microplate Luminometer (Promega) or a plate reader-SpectraMax iD3 (Molecular Devices, San Jose, CA, USA). The firefly luciferase activities were normalized to the Renilla luciferase activities. 

### 2.8. Cell Counting Assay

A549 and H2030 cells cultured in RPMI 1640 containing 5% FBS in 12 well-plates at 20% confluence were treated with Oxy210 for 6 days and then trypsinized, spun down and resuspended in fresh medium. For the Carboplatin (CP) and Oxy210 co-treatment experiments, A549 cells cultured in RPMI 1640 containing 2% FBS in 12 well-plates at 20% confluence were treated with CP at different concentrations in the presence or absence of Oxy210 for 6 days. For the TGFβ-induced drug resistance assay, A549 cells cultured in RPMI 1640 containing 2% FBS in 12 well-plates at 10% confluence were pre-treated with TGFβ1 alone or together with Oxy210 for 3 days, and then treated with 20 μM CP for 4 days. An aliquot of cell suspension was applied to a hemocytometer and the cells were then counted under a light microscope. 

### 2.9. Matrigel Invasion Assay

Invasion assays were performed using 24-well Matrigel invasion chambers (Corning, Corning, NY, USA), with the protocol provided by the manufacturer. A549 cells in 6-well plate starved in RPMI 1640 containing 0.1% FBS for 24 h were pre-treated with Oxy210 or DMSO for 2 h. 2 × 10^4^ cells were seeded in each of the upper chambers containing RPMI 1640 supplemented with 0.1% FBS and the test agents. Each of the lower chambers contained RPMI 1640 supplemented with 5% FBS and the corresponding test agents. The cells were cultured for 24 h before assessment of cell invasion. Non-invaded cells in the upper surface of the membrane were removed using a cotton swab and the invaded cells in the lower surface were fixed and stained with 100% methanol and 0.4% trypan blue, respectively. The invaded cells were imaged and counted under the microscope at 40X total magnification.

### 2.10. Western Blotting

A549 cells were pretreated with DMSO, Oxy186 (10 μM) or Oxy210 (10 μM) for 3 h, then treated with TGFβ (4 ng/mL) for the indicated times. After treatment, the cells were harvested in Triton lysis buffer (1% Triton X-100, 25 mM Tris-HCl, pH 7.4, 300 mM NaCl with protease and phosphatase inhibitors), and the supernatant was collected as cell lysates after centrifugation (15,000× *g*, 25 min at 4 °C). A Bradford assay was used to measure the protein concentration. 10 ng proteins of each sample were loaded into the wells of an 8% SDS-PAGE gel, and the proteins were transferred to a PVDF membrane after SDS-PAGE gel separation. The membrane was blocked with 5% milk for 1 h at room temperature and then incubated at 4 °C with appropriate dilutions of primary antibody (pSMAD2: 1:1,000, Cell Signaling Technology, Danvers, MA, USA #3108; pSMAD3, 1:5,000, Rockland Immunochemicals Inc., Pottstown, PA, USA #600-401-919; SMAD2, 1:1,000, Abcam, Cambridge, UK #ab63576; SMAD3, 1:1,000, Abcam #ab40854; HSC70, 1:10,000, Santa Cruz Biotechnology, Dallas, TX, USA #sc-7298. After washing steps, the membrane was incubated with horseradish peroxidase conjugated secondary antibody with 1:5,000 dilution (Cell Signaling Technology #7074 or #7076) at room temperature for 1 h. West Pico PLUS Chemiluminescent Substrate #34578 (Thermo Fisher Scientific, Waltham, MA, USA) was used to develop the signal, and the images were acquired using HyBlot films #E3018 (Denville Scientific Inc., Metuchen, NJ, USA) and a darkroom developer. Densitometry readings were performed with ImageJ software (Version 1.52a, NIH, Bethesda, MD, USA, see Supplementary Materials).

### 2.11. TGFβ Receptor Kinase Inhibition Assays

Effect of Oxy210 on inhibition of TGFβ receptors I and II kinase activity was assessed by Eurofins (Celle-L’Evescault, France). TGFBR1 (h): Human TGFBR1 (amino acid 200 to end) was incubated with 8 mM MOPS pH 7.0, 0.2 mM EDTA, 1 mM MnCl2, 2 mg/mL casein, 10 mM Magnesium acetate and [9-33P]-ATP (specific activity and concentration as required). The reaction was initiated by the addition of the Mg/ATP mix. After incubation for 40 min at room temperature, the reaction was stopped by the addition of phosphoric acid to a concentration of 0.5%. 10 μL of the reaction was then spotted onto a P30 filtermat and washed four times for 4 min in 0.425% phosphoric acid and once in methanol prior to drying and scintillation counting. TGFBR2 (h): Human TGFBR2 (amino acid 188-end) was incubated with 20 mM Tris/HCl pH 8.5, 0.2 mM EDTA, 0.33 mg/mL MBP, 10 mM Magnesium acetate and [9-33P-ATP] (specific activity and concentration as required). The reaction was initiated by the addition of the Mg/ATP mix. After incubation for 120 min at room temperature, the reaction was stopped by the addition of phosphoric acid to a concentration of 0.5%. 10 μL of the stopped reaction was spotted onto a P30 filtermat and washed four times for 4 min in 0.425% phosphoric acid and once in methanol prior to drying and scintillation counting [29].

### 2.12. Statistical Analysis

**Figure 4:**
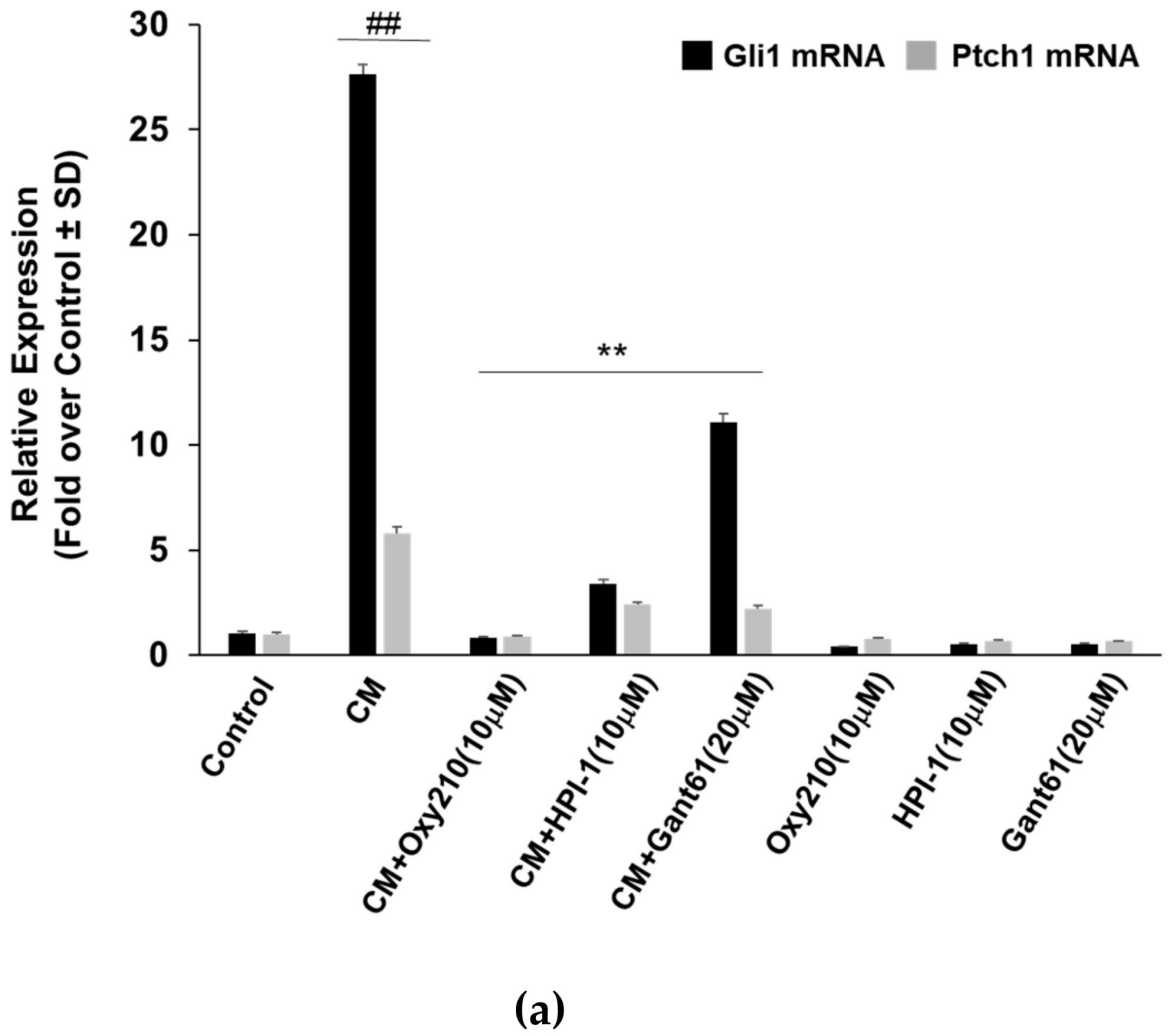

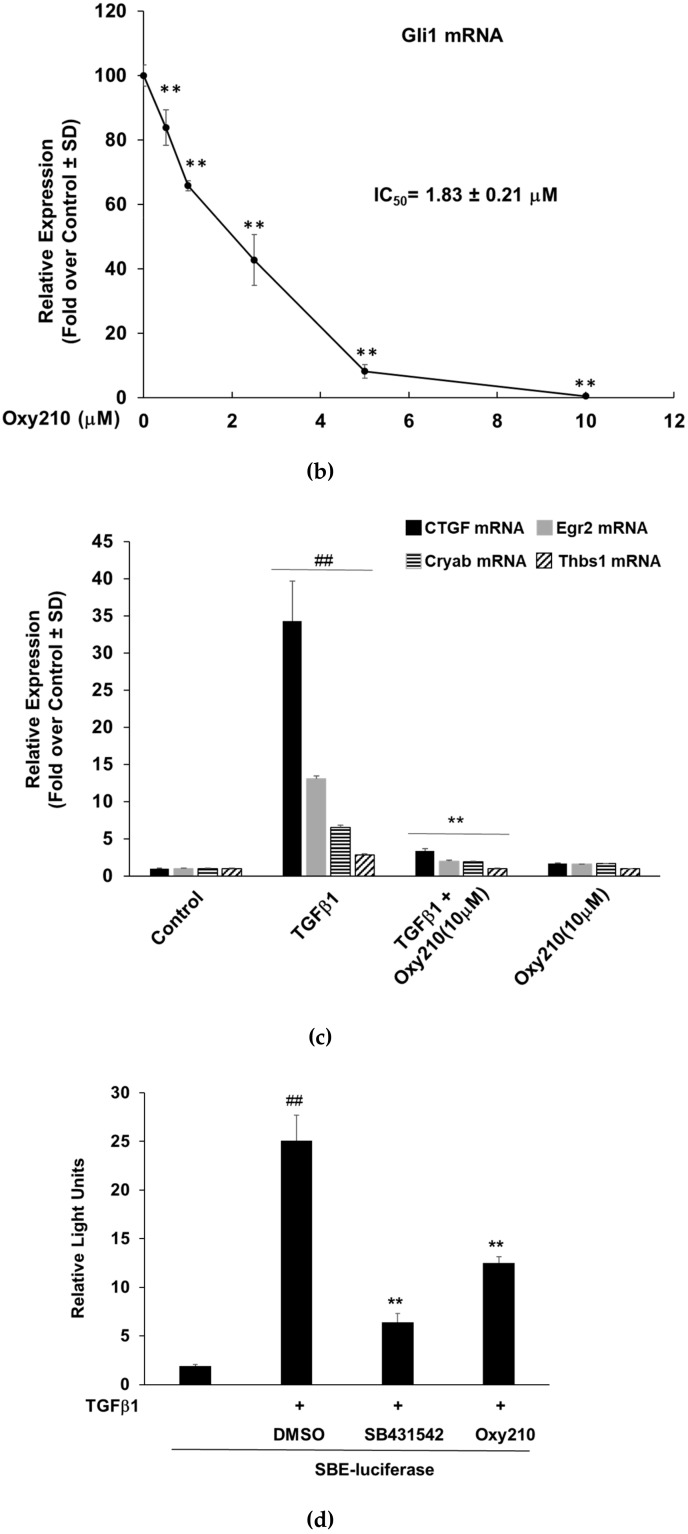

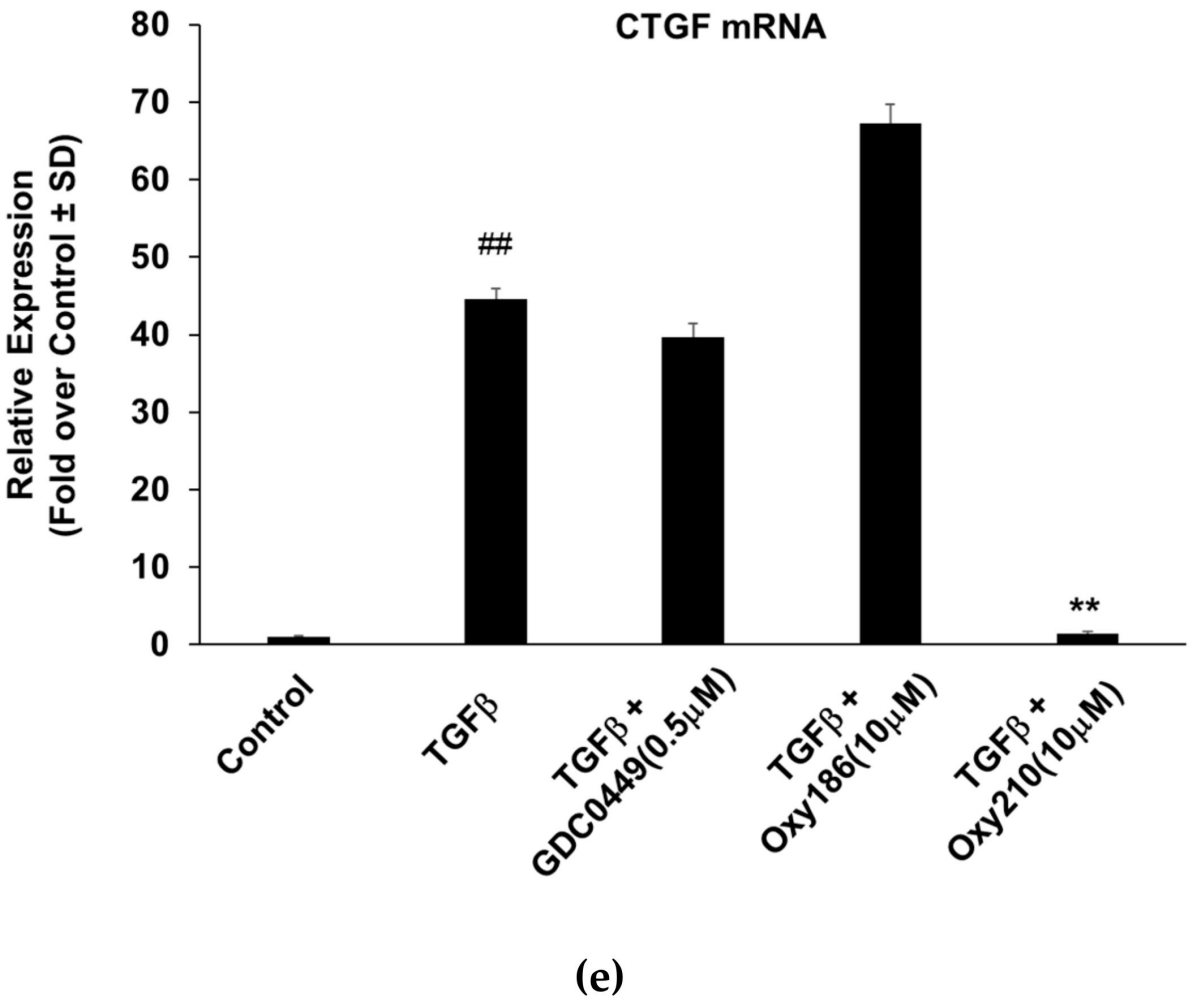
Inhibition of ligand-induced Hedgehog and TGFβ signaling in mouse fibroblast cells. NIH3T3 cells were pretreated for 2 h with Oxy210 or various inhibitors as indicated in DMEM containing 5% FBS. Next, cells were treated with CAPAN-1 conditioned medium (CM) in the absence or presence of oxysterols or inhibitors. After 72 h, RNA was extracted and analyzed by Q-RT-PCR for the expression of Hh target genes *Gli1* and *Ptch1* and normalized to Oaz1 expression (**a**,**b**). (**c**,**e**) NIH3T3 cells were treated in DMEM containing 0.1% BCS overnight and then pretreated for 2 h with the inhibitors as indicated in DMEM containing 0.1%BCS. The cells were then treated with TGFβ (20ng/mL) in the absence or presence of the compounds. After 48 h, RNA was extracted and analyzed by Q-RT-PCR for the expression of TGFβ target genes as indicated and normalized to Oaz1 expression. (**d**) NIH3T3 cells cultured in 24 well-plates were transfected with a Smad response element reporter (SBE-Luciferase) plasmid and a pTK-Renilla-Luciferase plasmid. 6 hrs after transfection, cells were treated with the test agents as indicated for 48 hrs. Luciferase activity was measured and normalized to the Renilla luciferase activity. Data from a representative experiment are reported as the mean of triplicate determinations ± SD (** *p* < 0.01 vs. CM (**a**,**b**) or TGFβ (**c**,**d** and **e**) treated cells; ## *p* < 0.01 vs. control).

Statistical analyses were performed using the StatView 5 program (SAS Institute, Cary, NC, USA). All p values were calculated using ANOVA and Fisher’s projected least significant difference (PLSD) significance test. A value of *p* < 0.05 was considered significant. The IC 50 dose-response curves shown in Figure 4b and Figure 7a–c were modeled using a five-parameter logistic model. This model allows for asymmetric curves and automatically estimates the mean maximum and minimum response. Based on this model, IC 50 values were estimated corresponding to the dose halfway between min and max response. Models of dose versus response and dose versus log response were also evaluated (see Supplementary Materials). The R square statistic was computed as a measure of model fit.

## 3. Results

### 3.1. Oxy210 Inhibits Hh and TGFβ Signaling in Mouse Fibroblast Cells

Having had identified the Hh pathway inhibitory effects of Oxy16 and Oxy186 [22,23], we examined the effects of Oxy210 on Hh signaling in NIH3T3-E1 mouse fibroblastic cells treated with CAPAN-1 conditioned medium (CM) that we previously reported to contain Hh ligands [22] and found that Oxy210 robustly inhibited the mRNA expression of ligand-induced Hh target genes Gli1 and Ptch1 (Figure 4a). Oxy210 exhibited strong inhibitory effects comparable to established Gli1 inhibitors, such as HPI-1 (10 μM) and Gant61 (20 μM), with an IC_50_ for inhibition of Gli1 expression of 1.83 ± 0.21 μM (Figure 4b). Interestingly, in addition to inhibiting Hh signaling, Oxy210 strongly inhibited TGFβ signaling in NIH3T3-E1 cells, as evidenced by the inhibition of the expression of TGFβ target genes, including Connective Tissue Growth Factor (CTGF), Early Growth Response 2 (Egr2), thrombospondin-1 (Thbs1) and crystallin, alpha B (Cryab) (Figure 4c). To further validate the inhibitory effect of Oxy210 on TGFβ signaling, we performed a luciferase reporter assay and found that Oxy210 and SB431542, an established TGFβ signaling inhibitor, significantly inhibited the TGFβ induced activity of SBE-luciferase reporter that is responsive to Smads in NIH3T3-E1 cells (Figure 4d). 

Since Gli1 has been reported to be involved in transcription of TGFβ target genes [16] and given the inhibitory effects of Oxy210 on Hh signaling, we sought to examine the possibility that Oxy210 could inhibit TGFβ target gene expression via suppression of Gli signaling. We examined the expression of CTGF induced by TGFβ in NIH3T3 cells treated with Oxy210, or Oxy186, a specific Hh pathway inhibitor that acts downstream of Smo [23], or GDC0449, a Smo antagonist, and found that only Oxy210 robustly inhibited the expression of CTGF (Figure 4e), suggesting that the anti-TGFβ signaling activity of Oxy210 is a unique feature that is independent of its anti-Hh signaling activity. It is noteworthy that the concentrations of Oxy210, Oxy186 and GDC0449 used in this study were previously confirmed to inhibit Gli-mediated Hh signaling in NIH3T3 cells. 

### 3.2. Oxy210 Inhibits Hh Signaling Downstream of Smo

**Figure 5:**
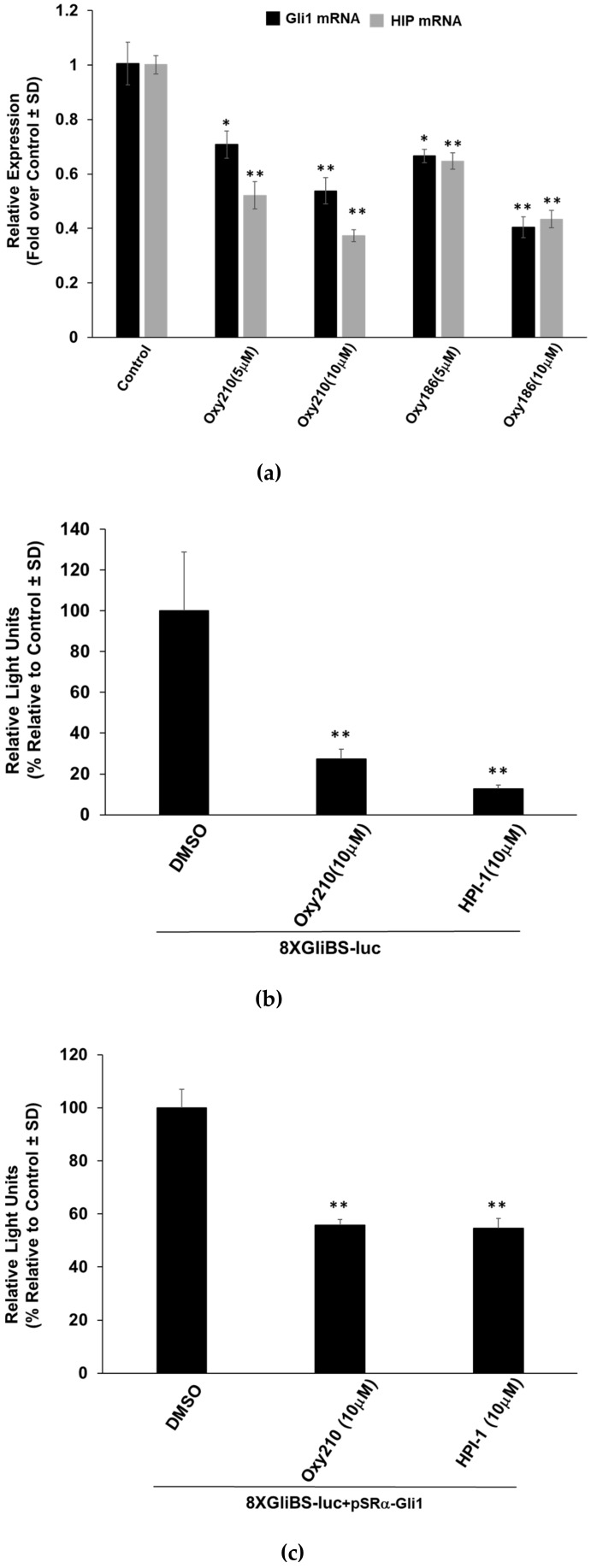
Oxy210 acts downstream of Smo. (**a**) Sufu-/- MEF cells were treated with DMEM containing 5% FBS in the presence or absence of Oxy210. After 72 h, RNA was extracted and analyzed by Q-RT-PCR for the expression of Hh target genes Gli1 and Hip, normalized to Oaz1 expression. (**b**) Sufu-/- cells cultured in 24 well-plate were transfected with a Gli response-element reporter (pGL3b-8xGliBS-Luciferase) plasmid and a pSV40-Renilla-Luciferase plasmid. 6 hrs after transfection, cells were treated with the compounds as indicated for 72 hrs. Luciferase activity was measured and normalized to the Renilla luciferase activity. (**c**) NIH3T3 cells cultured in 24 well-plate were transfected with a Gli response-element reporter (pGL3b-8xGliBS-Luciferase) plasmid, a pSV40-Renilla-Luciferase plasmid and a vector expressing Gli1, pSRa-Gli1. 6 h after transfection, cells were treated with the test agents as indicated for 72 hrs. Data from a representative experiment are reported as the mean of triplicate determinations ± SD (* *p* < 0.05 vs. DMSO or control; ** *p* < 0.01 vs. DMSO or control).

Suppressor of fused (Sufu) is a negative regulator of Hh signaling required to process Gli transcription factors and its loss results in a Smo-independent activation of Gli [30]. To examine whether Oxy210 inhibits Hh signaling epistatic to Sufu, we tested the effect of Oxy210 on Sufu-/- mouse embryonic fibroblasts (MEFs). Treatment of Sufu-/- MEFs with Oxy210 showed significant inhibition of high baseline expression of Hh pathway target genes Gli1 and Hip, suggesting that Oxy210, at least in part, exerts its inhibitory effect on Hh signaling epistatic to Sufu (Figure 5a). Furthermore, Oxy210 also significantly inhibited the activity of a Gli responsive luciferase reporter in Sufu-/- cells, in which the reporter was trans-activated by endogenous Gli that is constitutively activated due to the loss of Sufu (Figure 5b). HPI-1, a known Gli inhibitor, also inhibited the reporter activity (Figure 5b). Moreover, we examined the ability of Oxy210 to inhibit Gli1 transcriptional activity in NIH3T3-E1 cells and found that Oxy210 at 10 μM significantly inhibited the transcriptional activity of a Gli responsive luciferase reporter that was trans-activated by over expressing exogenous Gli1, comparable to HPI-1 at the same concentration (Figure 5c). Altogether, these findings suggest that Oxy210 inhibits Hh signaling, to a significant extent, downstream of Smo, unlike most other antagonists of the pathway, including the FDA approved drugs vismodegib, sonidegib and glasdegib that are known to block Hh signaling by antagonizing Smo [31,32].

### 3.3. Oxy210 Inhibits Expression of Glis and SHH in NSCLC Cells and Proliferation of NSCLC Cells 

**Figure 6:**
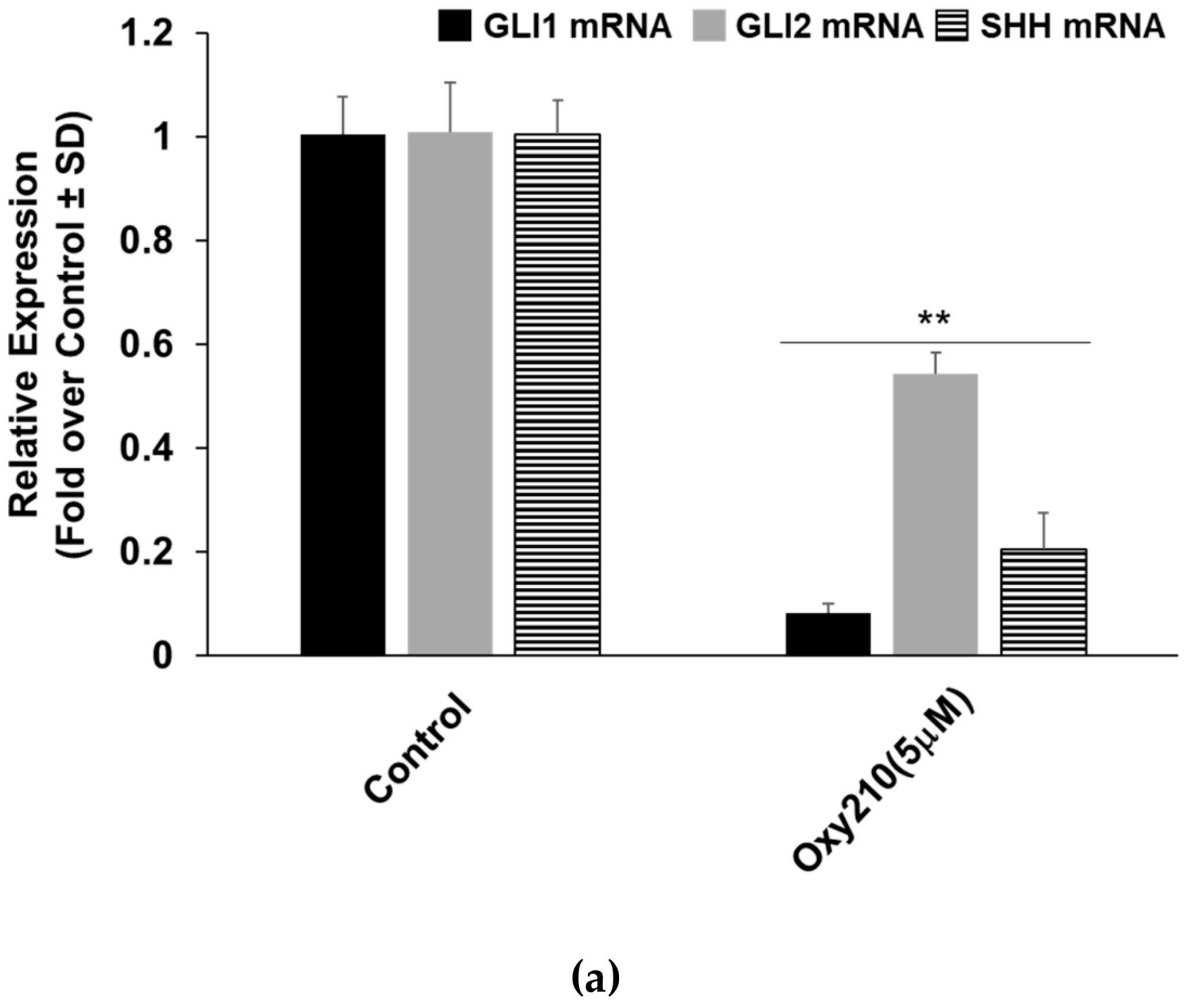

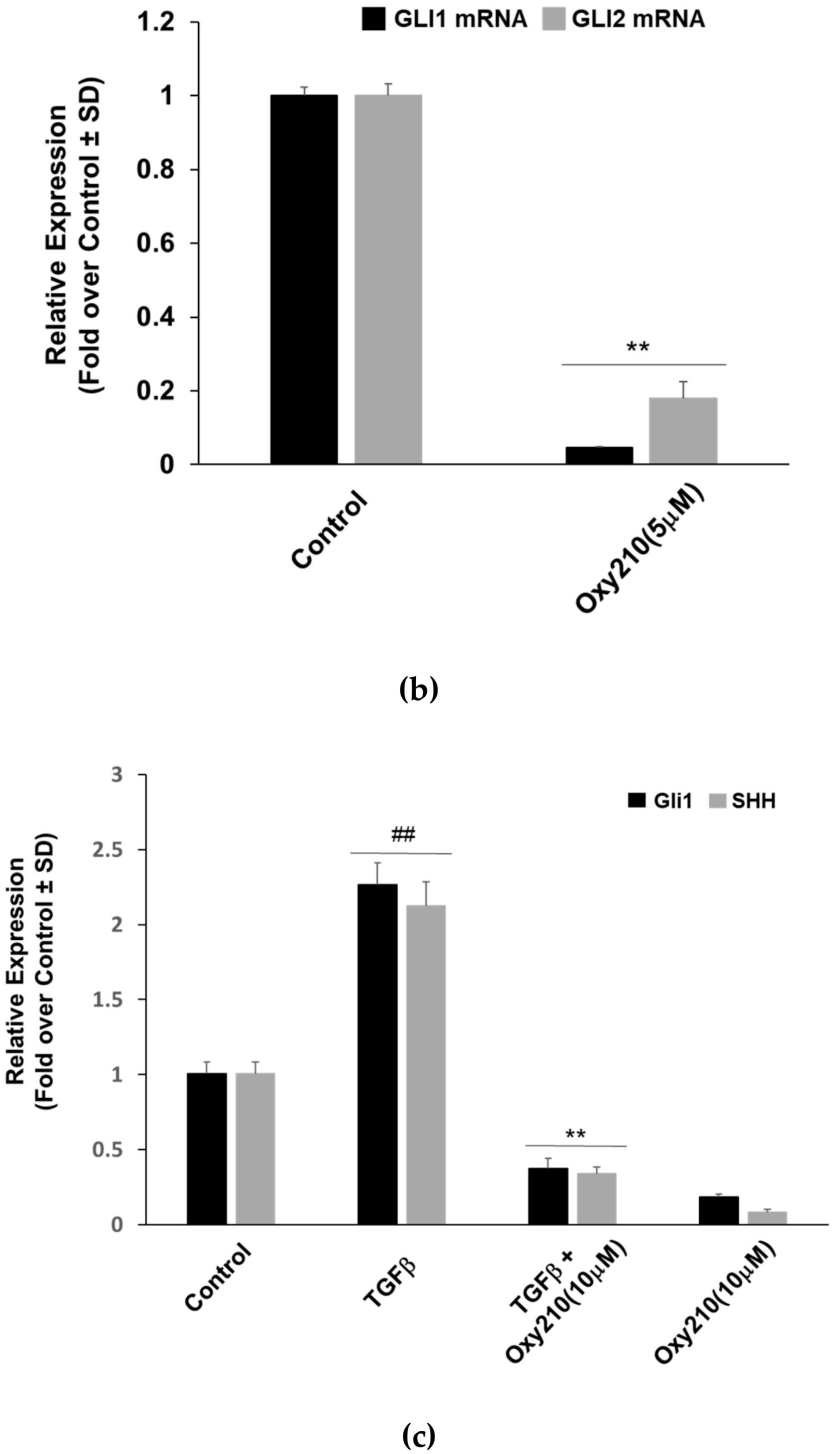
Inhibition of Glis and SHH expression in Non-Small Cell Lung Cancer (NSCLC) cells. A549 (**a**) and H2030 (**b**) cells were treated with Oxy210 in RPMI 1640 containing 5% FBS for 72 h, then RNA was extracted and analyzed by Q-RT-PCR for the expression of the indicated genes and normalized to *GAPDH* expression. (**c**) A549 cells were pretreated for 2 h with Oxy210 in RPMI 1640 containing 5% FBS. Next, cells were treated with TGFβ (10 ng/mL) in the absence or presence of Oxy210. After 48 h, RNA was extracted and analyzed by Q-RT-PCR for the expression of *Gli1* and *Shh* and normalized to *GAPDH* expression. Data from a representative experiment are reported as the mean of triplicate determinations ± SD (** *p* < 0.01 vs. TGFβ treated cells; ## *p* < 0.01 vs. control).

It has been demonstrated that Gli activity in many human malignancies, including small-cell lung cancer (SCLC) and NSCLC, is required for tumorigenesis [33]. Direct inhibition of Gli, perhaps in combination with other chemotherapeutic agents, could represent a promising strategy for the treatment of different malignancies, including NSCLC. We found that Oxy210 at 5 μM significantly inhibited the mRNA expression of GLI1 and GLI2 in both A549 and H2030 NSCLC cells (Figure 6a,b). In addition, TGFβ signaling has been reported to activate the Hh pathway via directly inducing GLI2 transcription, which in turn upregulates GLI1, in several types of cells [16,33,34]. We found that the expression of GLI1 and SHH mRNA was induced by TGFβ and that this induction was completely inhibited by Oxy210 in A549 cells, suggesting that Oxy210 can block the crosstalk between the two signaling pathways (Figure 6c). Moreover, tumor cells have been shown to produce Hh ligands that may stimulate tumor stromal cells to produce various factors that facilitate growth and metastasis of the tumor cells. This type of paracrine Hh signaling emanating from tumor cells is believed to play an important role in tumor growth and dissemination in several solid tumors, including NSCLC [33,34]. A549 cells produce SHH and CM harvested from these cells induces Hh signaling when applied to NIH3T3 cells (Parhami et al., unpublished observations). Oxy210 significantly inhibited the expression of SHH mRNA in A549 cells (Figure 6a), suggesting that Oxy210 could block paracrine Hh signaling by targeting both cancer cells and stromal cells. To the extent that the expression of SHH by tumor cells may stimulate fibroblasts and other non-cancerous cells in the tumor microenvironment, its inhibition by Oxy210 could allow for targeting Hh signaling in tumor cells as well as cells in the microenvironment. However, we do not mean to imply that SHH expression is a read out of Hh pathway activation or Gli signaling, except perhaps in some tumor cells with highly dysregulated signaling. 

**Figure 7:**
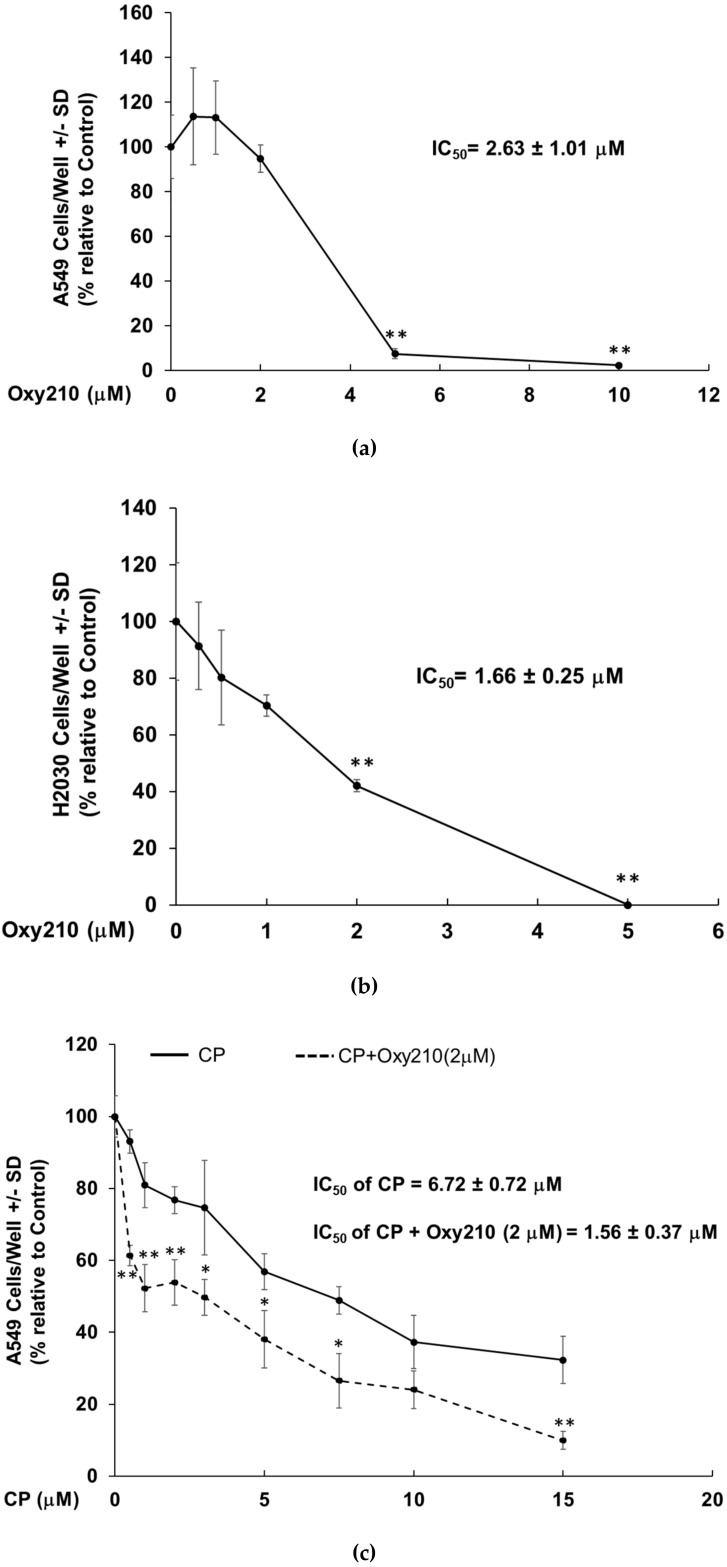
Inhibition of NSCLC cell proliferation by Oxy210. A549 (**a**), (**c**) and H2030 (**b**) cells were treated with the test agents as indicated in RPMI 1640 containing 5% FBS for 5 days and then were trypsinized and counted as described in Methods and Materials section. Data from a representative experiment are reported as the mean of triplicate determinations ± SD (* *p* < 0.05 vs. control; ** *p* < 0.01 vs. control).

Several reports have linked Hh signaling to upregulation of cell proliferation in lung cancer cells [33,34]. Accordingly, we investigated whether Oxy210 could inhibit cell proliferation of A549 and H2030 cells, using cell counting assays. As shown in Figure 7a,b, Oxy210 exerted significant anti-proliferative effects in A549 and H2030 cells, with an IC_50_ of 2.63 ± 1.01 μM and 1.66 ± 0.25 μM, respectively. In addition, Oxy210, when combined with non-cytotoxic concentrations of CP (1 to 10 μM) in A549 cells, significantly improved the anti-proliferation potential of CP (Figure 7c). Oxy210 reduced the IC_50_ of CP about four-fold from 6.72 ± 0.72 μM to 1.56 ± 0.37 μM in the presence of 2 μM Oxy210 (Figure 7c). 

### 3.4. Oxy210 Inhibits TGFβ-Induced EMT in NSCLC Cells

**Figure 8:**
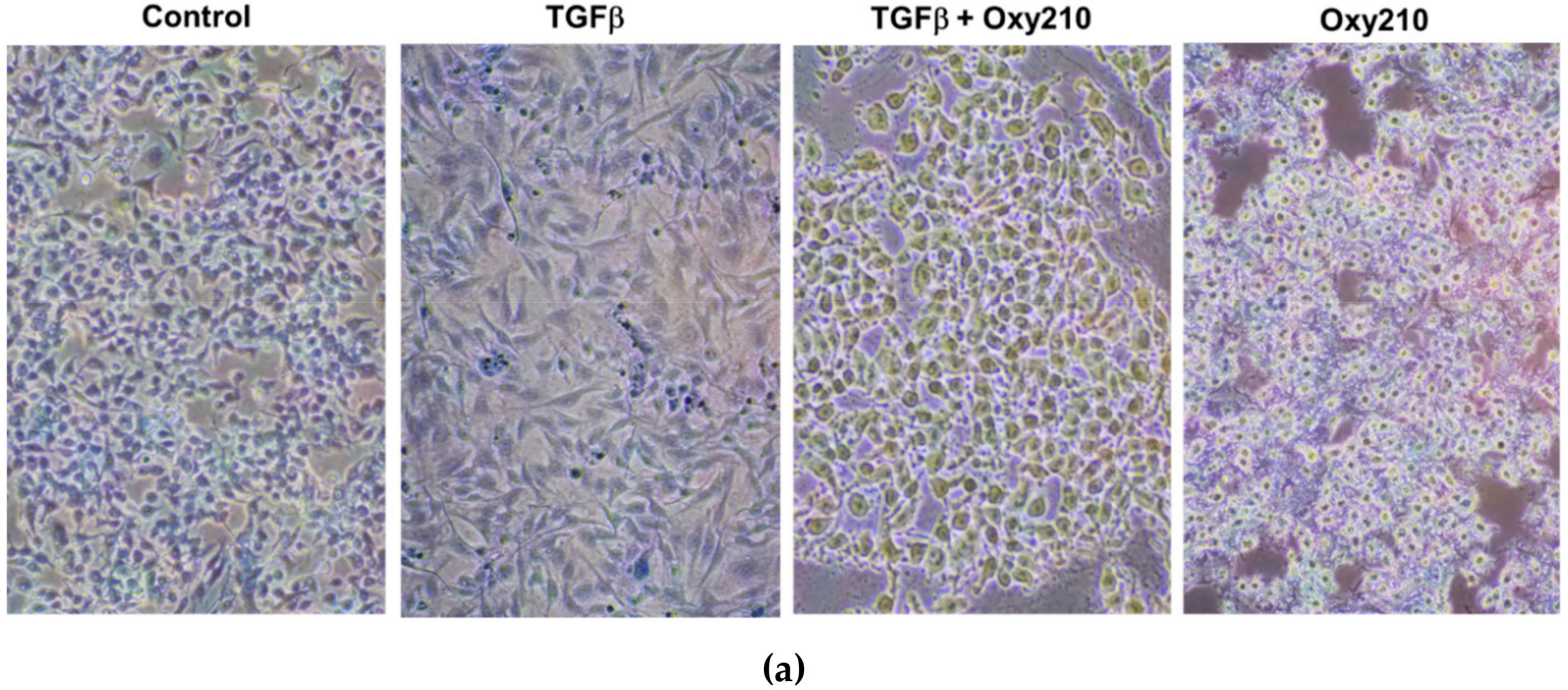

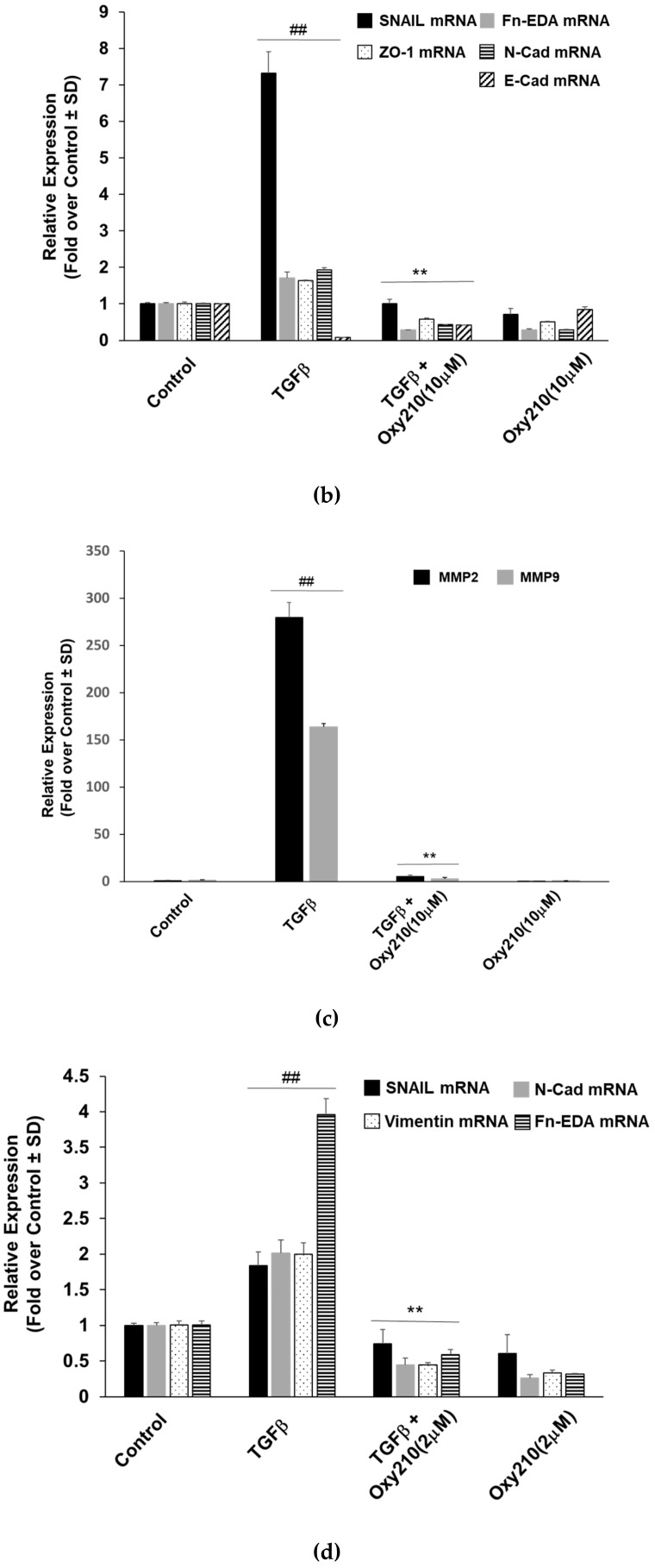
Inhibition of TGFβ-induced EMT in NSCLC cells. (**a**) A549 cells were treated with TGFβ in the absence or presence of Oxy210 in RPMI 1640 containing 2% FBS for 5 days. The cells were washed with PBS and then fixed with 2% formaldehyde in PBS at room temperature for 15 min. Formaldehyde was then removed, cells washed with PBS and stained with 4% Trypan Blue for 5 min. Phase contrast images were obtained using a 20X objective lens. A549 (**b**,**c**) and H2030 (**d**) cells were treated in RPMI 1640 containing 0.1% FBS overnight and then pretreated for 2 h with Oxy210 in RPMI 1640 containing 0.1% FBS. Next, cells were treated with TGFβ in the absence or presence of Oxy210. After 48 h, RNA was extracted and analyzed by Q-RT-PCR for the expression of the genes as indicated and normalized to *GAPDH* expression. Data from a representative experiment are reported as the mean of triplicate determinations ± SD (** *p* < 0.01 vs. TGFβ treated cells; ## *p* < 0.01 vs. control).

Emerging evidence suggests that EMT in NSCLC can be induced by TGFβ, increasing chemoresistance and invasive properties, and conveying stem cell-like characteristics to tumor cells [1,35]. Since it has been reported that A549 cells readily undergo phenotypic changes consistent with EMT upon exposure to TGFβ [36], we chose this cell line to examine the effects of Oxy210 on EMT. After 7 days of exposure to TGFβ, A549 cell morphology changed to a mesenchymal phenotype, with an elongated and disseminated appearance (Figure 8a) that was accompanied by upregulation of mRNA expression for genes involved in EMT including SNAIL, ZO-1, Fn-EDA and N-Cadherin (*N*-Cad) and downregulation of of the epithelial gene E-Cadherin (E-Cad) (Figure 6b). Oxy210 inhibited the expression of mesenchymal genes upregulated by TGFβ, partially rescued the expression of E-Cad downregulated by TGFβ (Figure 8b) and reversed the TGFβ-induced EMT morphology changes outlined earlier (Figure 8a). Matrix metalloproteinases (MMPs) stimulate tumorigenesis, cancer cell invasion and metastasis by degrading and modifying the extracellular matrix (ECM) as well as cell-ECM and cell-cell contacts, facilitating detachment of tumor cells from the surrounding tissue [37]. Oxy210 inhibited the mRNA expression of MMP2 and MMP9 that was robustly induced by TGFβ in A549 cells (Figure 8c). In addition, we further studied the inhibitory effects of Oxy210 on EMT in another NSCLC cell line, H2030, and found complete suppression of genes associated with TGFβ-induced EMT by Oxy210 in these cells (Figure 8d). 

### 3.5. Oxy210 Inhibits TGFβ-Induced Invasive Activity of A549 Cells 

**Figure 9:**
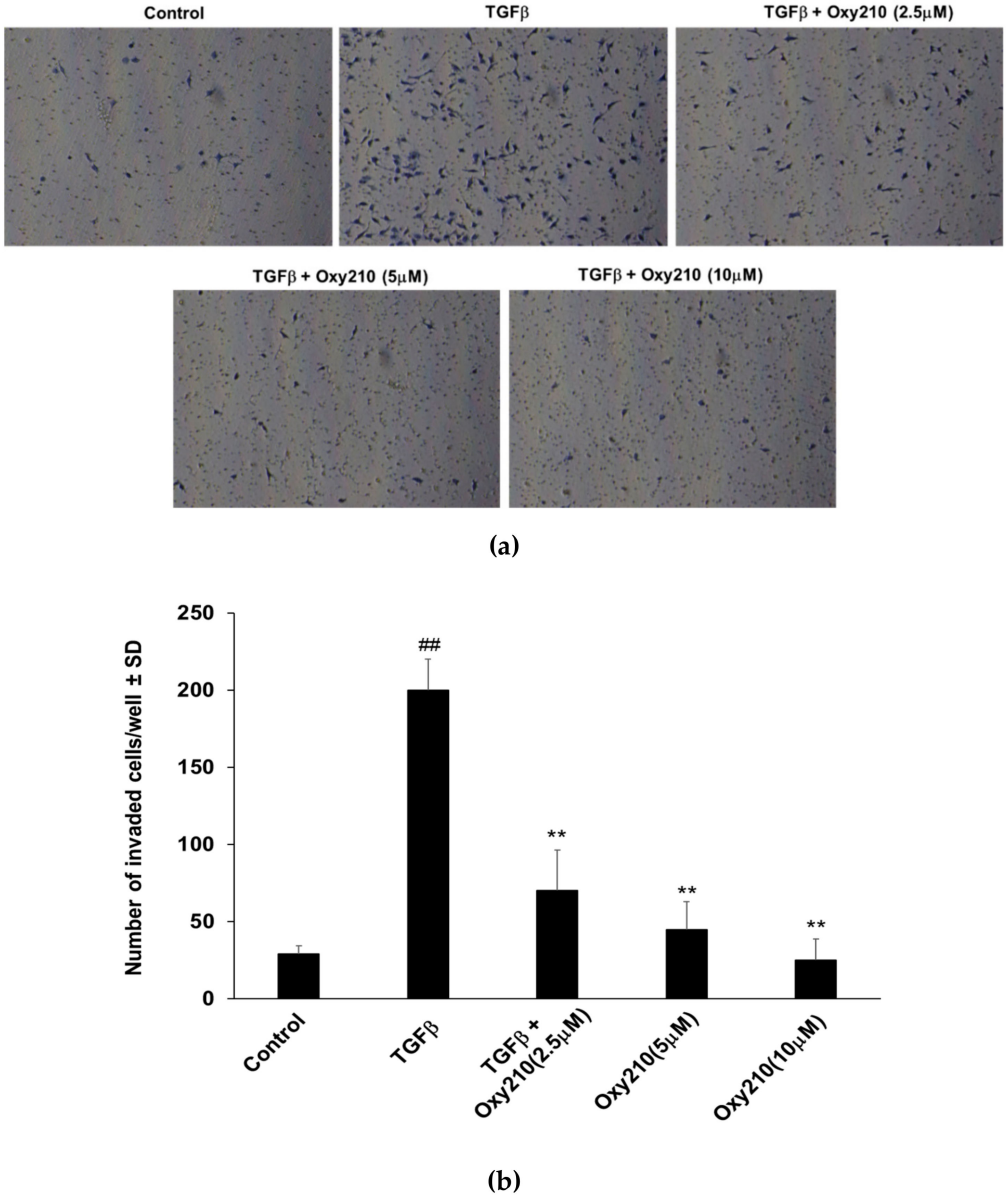
Inhibition of TGFβ-induced invasive activity of A549 cells. In vitro invasion assays were performed using chambers with matrigel-coated membrane as described in the Materials and Methods section. (**a**) Images of invaded cells on the membranes. (**b**) Quantitative analysis of invasion of A549 cells induced by TGFβ in the absence or presence of Oxy210. Data from a representative experiment are reported as the mean of triplicate determinations ± SD (** *p* < 0.01 vs. TGFβ treated cells; ## *p* < 0.01 vs. control).

EMT is associated with cell invasion, metastasis and disease progression in NSCLC [35]. Using an in vitro invasion assay, we investigated if Oxy210 can inhibit the invasive activity of A549 cells that have undergone TGFβ-induced EMT. We found that TGFβ strongly enhanced the invasive activity of A549 cells and that Oxy210 inhibited this invasive activity in a dose dependent manner (Figure 9a,b). These observations are consistent with the downregulation of EMT by Oxy210 in A549 cells described earlier.

### 3.6. Oxy210 Inhibits Drug-Resistance Associated with TGFβ-Induced EMT in NSCLC Cells 

**Figure 10:**
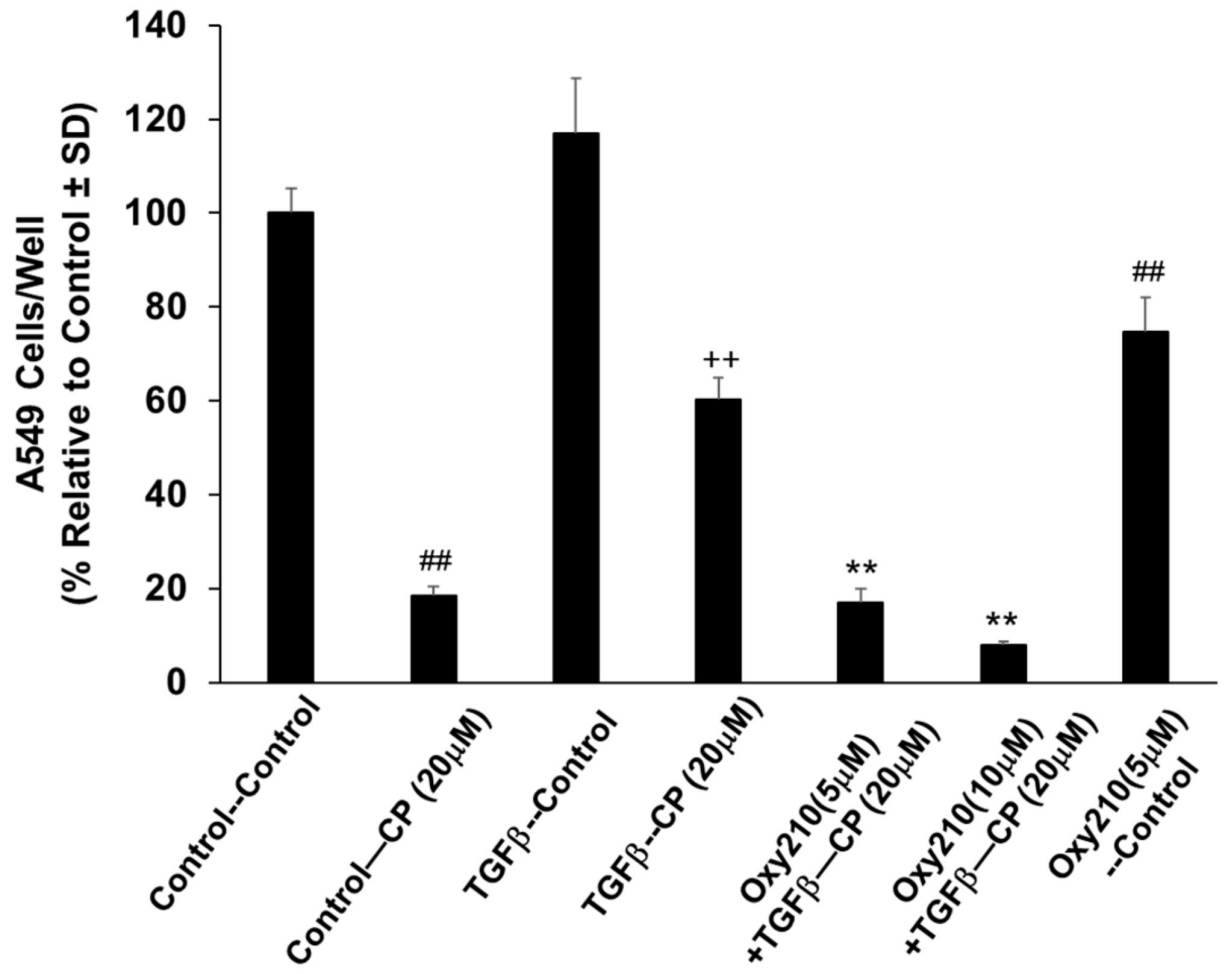
Effect of Oxy210 on TGFβ-induced resistance to carboplatin. A549 cells were treated with the agents as indicated (in front of --) in RPMI 1640 containing 2% FBS for 3 days, and then treated with the agents as indicated (after --) for 4 days. The cells were then trypsinized and counted. Data from a representative experiment are reported as the mean of triplicate determinations ± SD (## *p* < 0.01 vs. Control--Control; ++ *p* < 0.01 vs. Control--CP; ** *p* < 0.01 vs. TGFβ--CP).

In advanced NSCLC, activated TGFβ signaling is a key mediator of drug resistance, including therapy induced drug resistance, with complex mechanistic features. TGFβ signaling is a major driver of EMT in NSCLC cells, a cellular transformation that facilitates drug resistance to cytotoxic agents such as platin based as well as targeted therapies and immunotherapy [35,36]. These features make TGFβ inhibition a target for combination therapies that may improve treatment responses and delay the onset of drug resistance in NSCLC patients. We found that A549 cells pre-treated with TGFβ1 for 3 days were rendered significantly less sensitive to CP, while cells pre-treated with TGFβ1 together with Oxy210 showed similar sensitivity to that observed in cells treated with CP alone suggesting that Oxy210 has the potential to inhibit the CP-resistance induced by TGFβ1 in A549 cells (Figure 10). 

### 3.7. Oxy210 Inhibits Phosphorylation of SMAD2 and SMAD3 Induced by TGFβ

**Figure 11:**
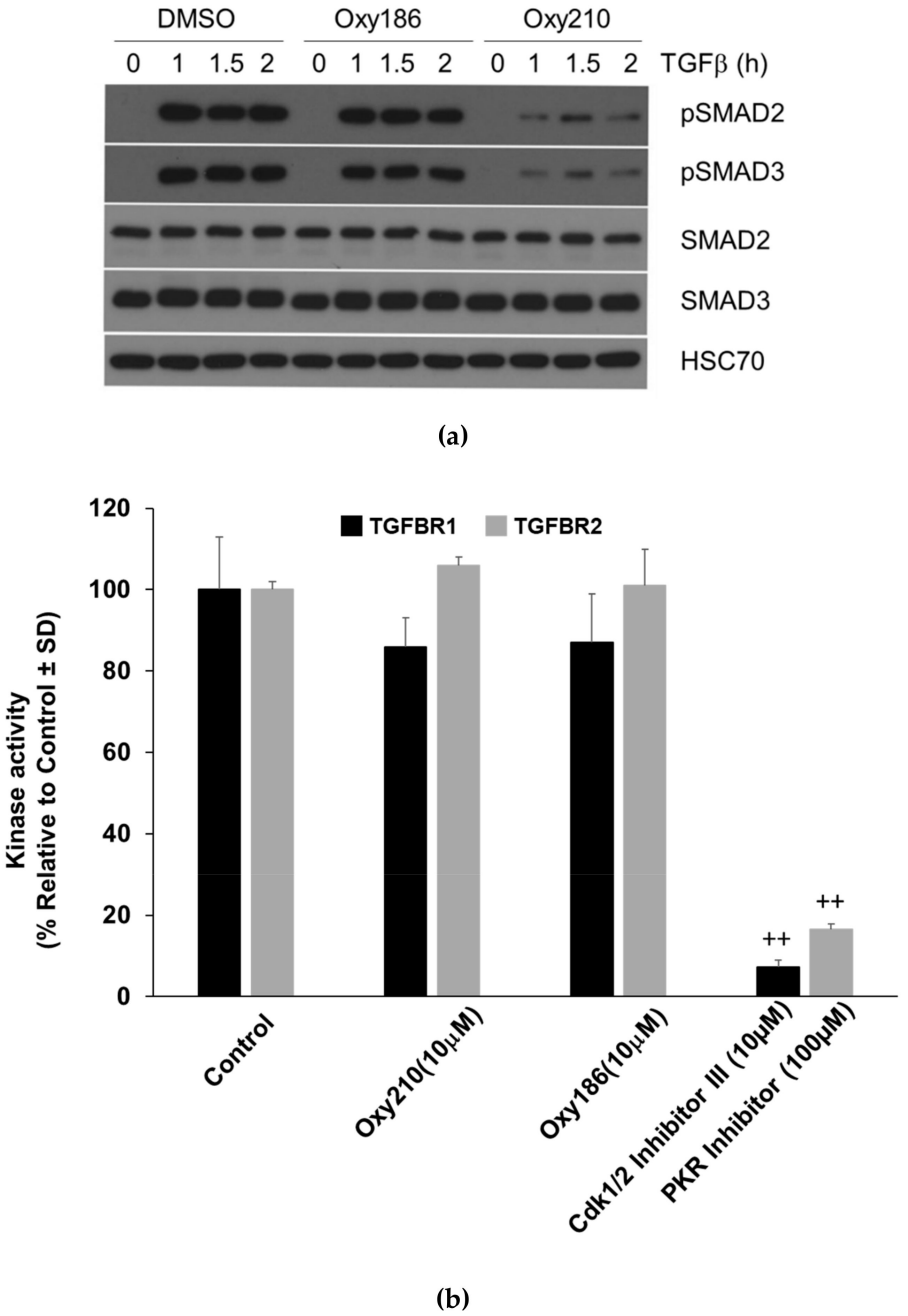

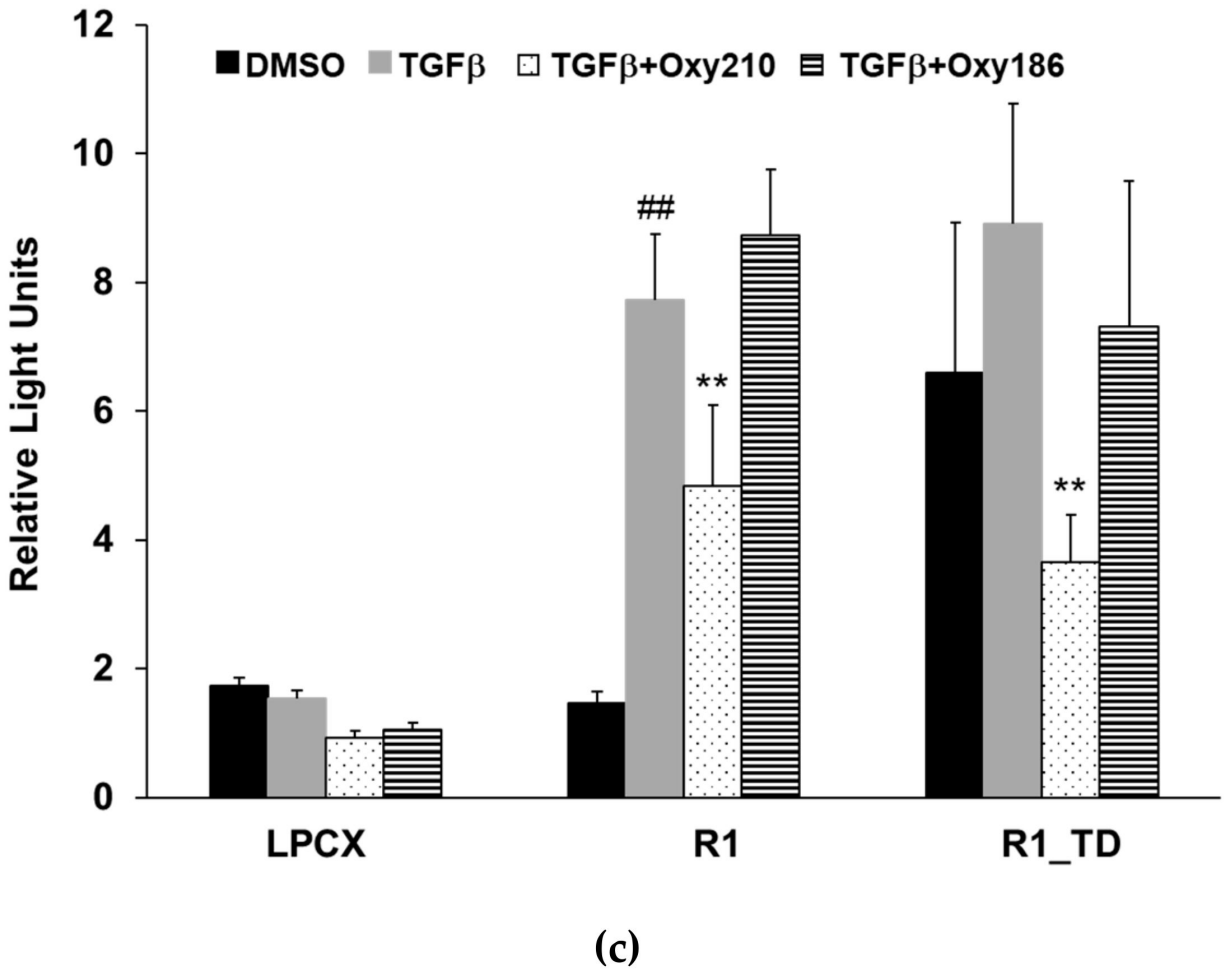
Inhibition of TGFβ-induced Smad2/3 phosphorylation and transcriptional activity. (**a**) Western blot analysis of A549 cells. Cells were pretreated with DMSO, Oxy186 or Oxy210 for 3 h, then treated with TGFβ (4 ng/mL) for the indicated times. (**b**) Kinase assay was performed as described in Materials and Methods with the agents as indicated. (**c**) TGFβRI-deficient R1BL17 cells were transfected with TGFβRI plasmid (WT or TD mutant), Smad responsive luciferase reporter (CAGA12-Luc), and pTK-Renilla-Luciferase plasmid. Twenty-four hours after transfection, cells were treated with the test agents as indicated for 20 hrs. Luciferase activity was measured and normalized to the Renilla luciferase activity. Data from a representative experiment are reported as the mean of triplicate determinations ± SD (** *p* < 0.01 vs. TGFβ treated cells; ## *p* < 0.01 vs. DMSO; ++ *p* < 0.01 vs. Control).

Upon binding TGFβ ligand, TGFβ type II receptor (TβRII), phosphorylates the TGFβ type I receptor (TβRI) that subsequently phosphorylates and activates downstream signal transducers called receptor-regulated Smads (R-Smads). R-Smad phosphorylation state determines Smad complex assembly/disassembly, nuclear import/export, transcriptional activity and stability, and is thus the most critical event in TGFβ signaling [38]. Smad2 and Smad3 are members of R-Smad that are phosphorylated and activated upon activation of TβRII and TβRI. We investigated if Oxy210 inhibits TGFβ signaling via inhibiting TGFβ-induced phosphorylation of Smad2 and Smad3. As shown in Figure 11a, TGFβ- induced phosphorylation of Smad2 and Smad3 was robustly inhibited by Oxy210 but not Oxy186 in A549 cells. Using Oxy186 as a negative control, we next investigated whether Oxy210 can inhibit the activity of TβRII and TβRI in functional kinase assays [29]. Surprisingly, Oxy210 (nor Oxy186) did not display significant inhibitory activity in these assays compared to Cdk1/2 Inhibitor III and PKR Inhibitor used as positive controls (Figure 11b). This suggests that, unlike SB431542 and clinical candidate galunisertib [39,40], Oxy210 does not act as a kinase inhibitor of TβRI or TβRII. We then investigated if Oxy210 inhibits the activity of TβRI using a luciferase reporter assay. R1BL17 cells, a mink lung epithelial cell line that does not express TβRI, were transiently transfected with a Smad responsive luciferase reporter and a vector (LPCX) expressing TβRI (RI) or expressing a constitutively active version of TβRI (RI_TD) or an empty LPCX and then treated with TGFβ in the presence of Oxy210 or Oxy186. As shown in Figure 11c, the activity of the reporter stimulated by either TGFβ-activated RI or ligand independent RI_TD was significantly inhibited by Oxy210 but not Oxy186, suggesting that Oxy210 acts at the level of activated TβRI and the Smad2/3 interaction.

### 3.8. Conformational Analysis of Oxy16, Oxy186 and Oxy210

**Figure 12:**
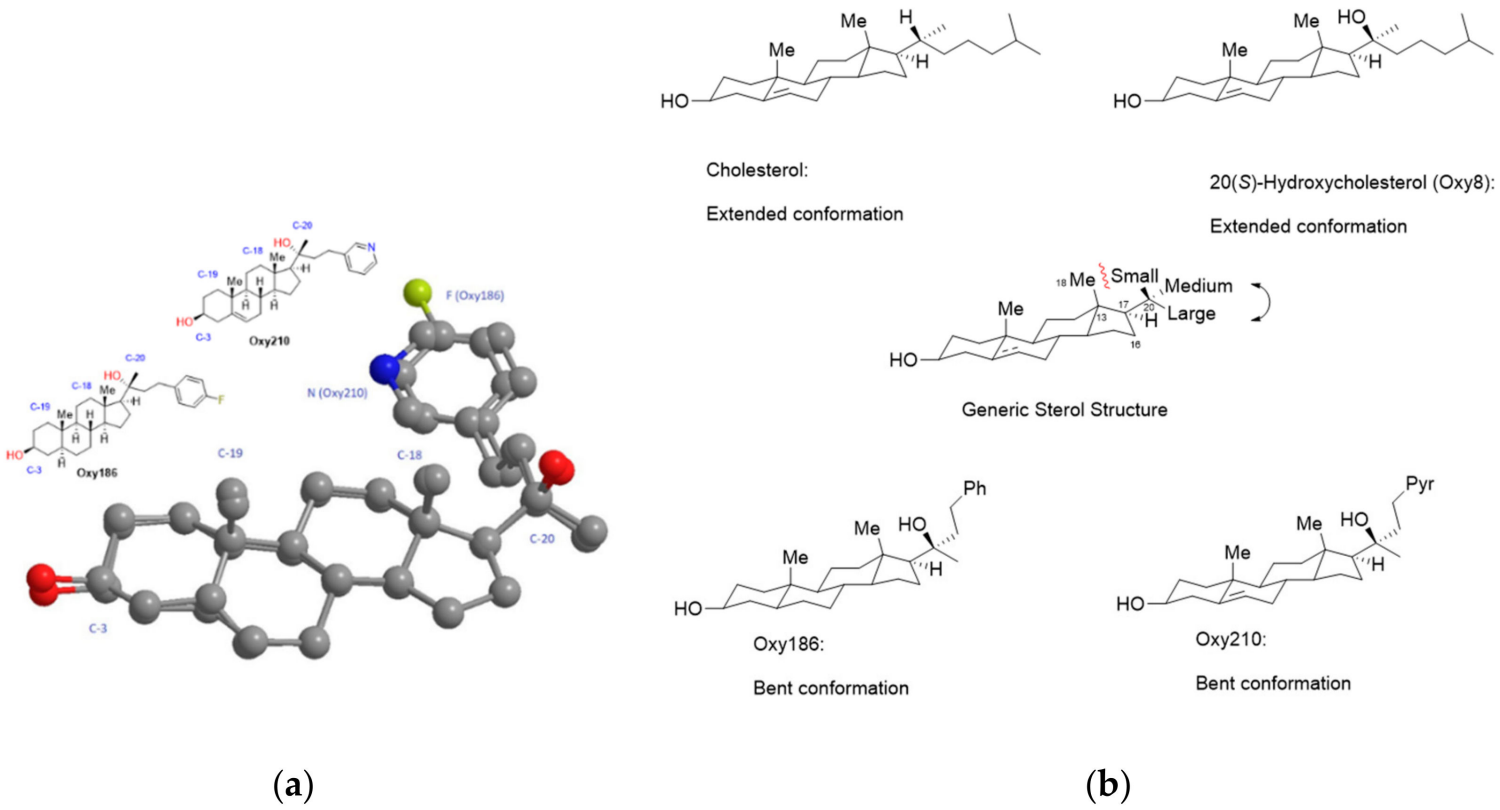
Conformational properties of Oxy186 and Oxy210 (**a**) The molecular structures of Oxy186 and Oxy210 and an overlay of their crystal structures. A structure alignment algorithm (Mercury, The Cambridge Crystallographic Data Centre) determined the minimal root mean square deviation (RMSD) of atomic positions in the overlay to equal 0.221 Å. (**b**) Varying the substitution pattern and stereochemical configuration of the C17–C20 bond, can result in a preference for either an extended or bent conformation of the ‘Large’ substituent, usually the sterol side, with respect to the tetracyclic sterol core.

To better understand the biological similarities and differences between Oxy16, Oxy186 and Oxy210 at a molecular level, single crystal x-ray diffraction studies were performed with these three molecules ([23], Figure 2 and Figure 3). In addition to a rigorous confirmation of structural identity, crystal x-ray diffraction studies can illuminate conformational properties in the solid state, including the overall shape of the molecules and orientation of the sterol side chain. As detailed in the Discussion section, Oxy16, Oxy186 and Oxy210, display solid state conformations in which the side chain is bent relative to their sterol core, rather than extended forward. This is in contrast to sterols and oxysterols with Smo binding affinity, such as cholesterol and Oxy8, a Hh pathway agonists, that display extended side chain conformations relative to their sterol core, both in the solid state and when bound to the Smo protein [41,42,43] (Figure 12b). However, by comparing the solid-state structures of Oxy186 and Oxy210, we cannot explain the ability of Oxy210 to inhibit both Hh and TGFβ signaling or explain why Oxy186 only inhibits Hh signaling.

### 3.9. Drug-Like Properties of Oxy210 

**Figure 13:**
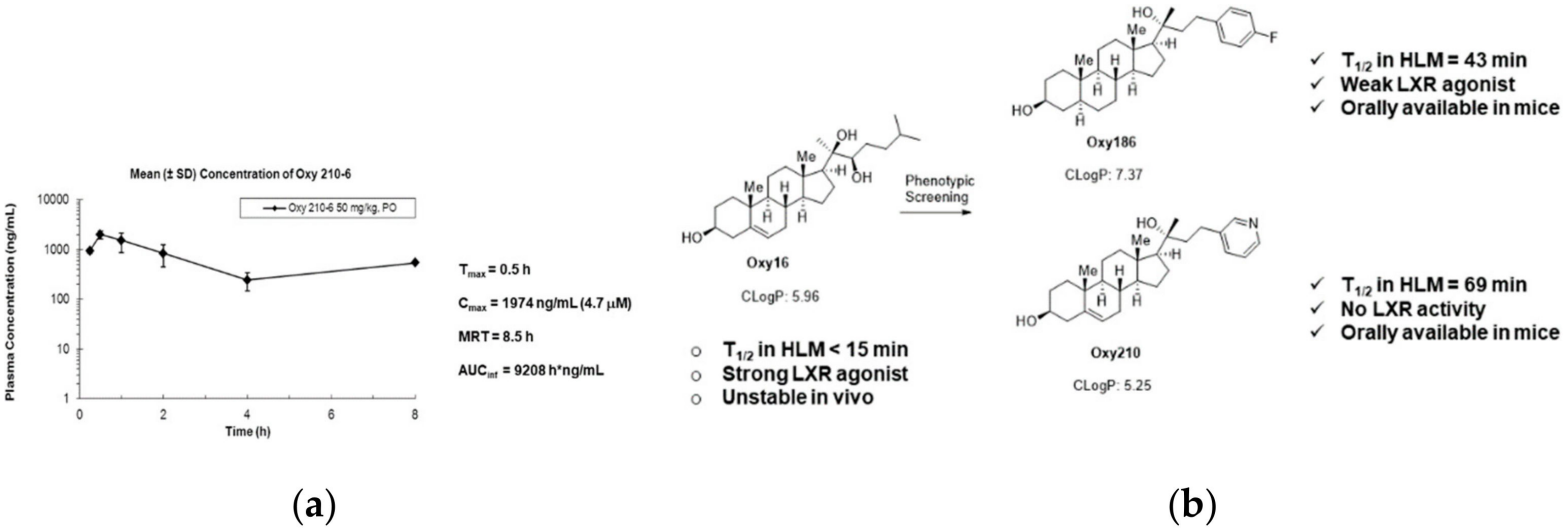
Drug-like properties of Oxy210. (**a**) A single dose of Oxy210 at 50 mg/kg, formulated in 3% DMSO + 7% Ethanol + 5% PEG400 + 85% corn oil, was administered to balb/c mice by oral gavage. Plasma samples were taken at 10 min, 30 min, 1h, 2h, 4h, 6h, and 8h, followed by LC/MS analysis of the plasma. (**b**) Evolution of Oxy210 and Oxy186 from parent compound Oxy16 and their drug-like properties.

In an initial assessment of drug-like properties of Oxy210, we performed a pharmacokinetic (PK) study in mice. Oxy210, formulated in 3% DMSO + 7% Ethanol + 5% PEG400 + 85% corn oil, was administered to balb/c mice by oral gavage as a single dose of 50 mg/kg. Plasma samples were taken at 10 min, 30 min, 1 h, 2 h, 4 h, and 8h, followed by LC/MS analysis. As shown in Figure 13a, Oxy210 displays attractive drug-like absorption and elimination characteristics, with the maximum serum concentration (Cmax) of 1974 ng/mL (4.7 μM) observed after 30 min (Tmax) and a mean residence time of 8 h. Additional PK studies in mice were performed with Oxy210 in a dose escalation study with repeated once daily oral dosing (50 mg/kg, 200 mg/kg and 400 mg/kg) for 7 days. Oxy210 was well tolerated by the mice, even at the highest dose of 400 mg/kg, and linear PK parameters and exposure were encountered during the study. Furthermore, Oxy210 was more stable than its parent compound, Oxy16, when incubated with human and mouse liver microsomes (HLM, MLM), as depicted in Table 1. Additionally, Oxy210 was found to be devoid of undesirable Liver X Receptor (LXR) activity, as determined by the lack of induced expression of SREB1c in HepG2 human liver hepatocytes, which is a mediator of liver steatosis, and the absence of ABCA1 expression in NIH3T3 cells. The drug-like properties of Oxy210 in comparison to Oxy16 are summarized in Figure 13b, suggesting that Oxy210 could be an attractive compound for further therapeutic development.

## 4. Discussion

In this report we describe the identification and characterization of new oxysterol-based dual inhibitors of TGFβ and Hh signaling, exemplified here by Oxy210, that could serve as potential drug candidates for indications associated with the aberrant activation of these signaling pathways. Specifically, we have studied these new inhibitors using in vitro assays that model cellular signaling relevant to human NSCLC and its tumor microenvironment: (1) inhibition of Hh and TGFβ signaling in NIH3T3-E1 fibroblasts treated with Hh ligands or TGFβ1, a model of paracrine Hh and TGFβ signaling; (2) inhibition of Hh signaling and proliferation of NSCLC cells, a model of tumor cell growth; and (3) inhibition of EMT induced by TGFβ and TGFβ-induced invasiveness of NSCLC cells, assays that model crucial and necessary steps observed during NSCLC metastasis; and (4) proliferation of NSCLC cells in the presence and absence of TGFβ ligand and CP; a model for drug resistance in lung cancer cells. To put the present report in the context of our past and ongoing efforts, we had previously examined oxysterol analogues in search of activators and inhibitors of Hh signaling, starting with naturally occurring metabolites of cholesterol, such as Oxy8 (20(*S*)-hydroxycholesterol) and Oxy16 (20α, 22(*R*)-dihydroxycholesterol), shown in Figure 1. We and others have characterized Oxy8 and related analogues, such as Oxy133, as Hh pathway agonists that act through allosteric activation of the Smo protein [25,45,46]. Separately, we reported that Oxy16 inhibits Hh signaling through an unknown mechanism downstream and independent of Smo [22,46]. Subsequently, we characterized Oxy186, a semisynthetic oxysterol analogue that mechanistically resembles Oxy16, as a significantly more potent inhibitor of Hh signaling acting downstream of Smo [23]. Both Oxy16 and Oxy186 (Figure 6e) are devoid of TGFβ inhibitory properties. 

Oxy186 and Oxy210 share close structural similarities and differ mainly in the aromatic portion of the sterol side chain where the 4-fluorobenzene moiety in Oxy186 is replaced with a 3-pyridyl group in Oxy210 (Figure 1). This suggests that a pyridyl group in the sterol side chain may be necessary for TGFβ inhibitory properties exhibited by Oxy210 but not for the Hh inhibitory properties shared by Oxy186 and Oxy210. Hence, the replacement of the hydrophobic 4-fluorobenzene with a more polar and weakly basic pyridine in the sterol side chain may act like a molecular switch that enables TGFβ inhibitory properties. The origins of this effect remain unidentified at this time but are likely connected to differences in physicochemical properties between Oxy186 and Oxy210. This notion is supported by the absence of any apparent differences between the physical shapes of the two molecules in the solid state, as demonstrated by overlapping crystal structures. In fact, Oxy186 and Oxy210 were determined to be ‘isostructural’ by x-ray diffraction analysis, sharing the same lattice system, space group and nearly identical unit cell parameters. In addition, the conformational properties displayed by Oxy186 and Oxy210 in the solid state are nearly identical and the two structures can be readily superimposed with low minimal root mean square deviation (RMSD) in the atomic positions of the overlay (RMSD = 0.221 Å), as illustrated in Figure 12a. The sterol side chains of Oxy186 and Oxy210 are both oriented in a U-shaped manner relative to the sterol core. Such conformational properties often correlate with (and may enable) the biological activities of sterols and oxysterols through shape complementarity with, and selective affinity for, protein receptors and sterol binding domains [47]. For example, Smo, the plasma membrane-associated signal transducer of Hh signaling features a sterol binding pocket located in an extracellular cysteine rich domain of the protein that controls the allosteric activation of the Hh pathway [45]. Sterol molecules with known Smo binding affinity, such as cholesterol, and oxysterol molecules that can allosterically activate Hh signaling in meaningful way, such as Oxy8, have been reported to display an extended conformation of the sterol side chain in the solid state and when bound to the Smo protein, according to detailed crystallographic and protein-crystallographic studies ([41,42,43] and Figure 12b). We have confirmed similar conformational properties for the Hh agonist, Oxy133, via x-ray diffraction analysis. By contrast, oxysterol molecules with Hh inhibitory properties, such as Oxy16, Oxy186 and Oxy210, display ‘bent’ or ‘curved’ overall shapes in the solid state, i.e., conformations in which the orientation of the sterol side chain is not linearly extended with respect the tetracyclic sterol core (Figure 2 and Figure 3, Figure 12a), suggesting that these oxysterols are unlikely Smo binders, short of significant conformational rearrangements of the sterol side chain. The side chain is conformationally the most flexible part of the otherwise rigid sterol molecules; however, the high degree of substitution across C-17, C18 and C-20 significantly reduces the conformational flexibility in many sterols due to steric crowding, as illustrated in Figure 12b. In particular, the orientation of the sterol side chain can be influenced by the absolute configuration at C-20, resulting in conformational preferences for either extended (e.g., Oxy8 and Oxy133) or bent conformations (Oxy186 and Oxy210). The solid-state conformation of Oxy16 is affected by intramolecular hydrogen bonding between the glycolic hydroxyl groups located at C-20 and C-22 in the sterol side chain (Figure 3). As a result, the sterol side chain in Oxy16 is bent to the side with respect to the sterol core, rather than extended forward, as observed in cholesterol and Oxy8 (Figure 12b). Beyond Hh signaling, Oxy16 functions as a key metabolite of cholesterol, involved in the rate limiting step of steroid biosynthesis catalyzed by the cholesterol side-chain cleavage enzyme, also known as P450scc or Cyp11a1. During protein crystallographic studies, Oxy16 was co-crystallized with the Cyp11a1 protein [48]; here, we report the first solid state structure of Oxy16 outside of a protein complex. In support of our argument, the conformation exhibited by Oxy16 in the solid-state structure, shown in Figure 3, is nearly identical to its conformation when bound to the Cyp11a1 enzyme (RMSD = 0.155 Å). Based on shape complementarity with the allosteric binding pocket of the Smo protein, therefore, Oxy16, unlike Oxy8 [43,45], is not a good fit for the binding pocket, which may explain its lack of detectable Smo binding affinity in a Smo binding assay [46]. 

We content that the biological properties of Oxy210 described here are specific inhibitory effects, by and large limited to Hh and TGF β signaling, rather than the result of nonspecific binding events and/or a pan inhibition of cellular signaling. In support of a specific molecular mechanism for Oxy210, we have examined many structural analogues and stereoisomers of Oxy210 for inhibition of Hh and/or TGFβ signaling, including the C-20 epimer of Oxy210, a 20(*S*)-tertiary alcohol, but found that only compounds that fit Oxy210′s stereochemical mold retain activity. In addition, Oxy210 did not inhibit other signaling pathways examined. For example, stimulation of NIH3T3-E1 or A549 cells with other growth factors or cytokines in the presence of Oxy210, such as EGF or Il-1β, did not result in significant inhibition of their respective target gene expression, fibronectin and IL-6, respectively. 

Experimentally, dual inhibition of TGFβ and Hh signaling compared to single inhibition of TGFβ or Hh signaling alone, can illuminate the degree and consequences of compensatory overlap that exists between the two pathways. When we activated the crosstalk between Hh and TGFβ signaling by stimulating A549 cells with TGFβ1 protein (Figure 6c), upregulation of GLI and Hh ligand (SHH) expression was observed. Oxy210 significantly reversed these effects, suggesting that Oxy210 can effectively block the crosstalk between the two signaling pathways. In addition, Oxy210 treatment significantly reduced the endogenous gene expression for SHH in A549 cells (Figure 6a), indicating that both the ligand production emanating from tumor cells as well as the stromal response in fibroblasts can be targeted by Oxy210, in a potentially synergistic manner. We plan to examine this possibility in future in vitro and in vivo studies. 

Not unlike Oxy186 [23], Oxy210 also displays anti-proliferative properties in NSCLC cells as a standalone agent, a likely consequence of Gli inhibition, rather than TGFβ inhibition (Figure 6a–c and Figure 7a,b). According to published reports [39], TGFβ protein has been shown to slow the proliferation of A549 cells and the inhibition of TGFβ signaling mediated by SB431542 does not lessen the proliferation of A549 cells, consistent to our own observations.

When we studied Oxy210 in combination with CP, a drug standard in the treatment of lung cancer, the addition of Oxy210 enhanced the anti-proliferative response to CP in A549 cells, expressed by a four-fold ‘left shift’ in the IC_50_ curve for CP (Figure 7c). Moreover, we tested Oxy210 in A549 cells pretreated with TGFβ protein, a model of EMT induced treatment resistance. Consistent with published reports for cisplatin [36], we found that such cells drastically lose their sensitivity toward CP, even in the presence of high concentrations (20 μM, Figure 10), presumably due to increased drug resistance in a mesenchymal state of the cells. However, co-administration of Oxy210 (5 μM) to the TGFβ pretreatment fully restored CP drug sensitivity to the A549 cells in these experiments, suggesting that TGFβ induced in vitro drug resistance toward CP can be effectively overcome by Oxy210. Upregulation of cellular TGFβ and Hh signaling can be observed in many cancers and often correlates unfavorably with prognoses and disease progression, even when such signaling alone is not sufficient to drive tumorigenesis [17,18,35]. The therapeutic inhibition of TGFβ signaling is currently under clinical investigation for multiple oncological indications, including NSCLC, following several different approaches [40,49]: TGFβ antibody treatment (Fresolimumab); inhibition of Smad phosphorylation with kinase inhibitors (Galunisertib); RNA silencing approaches (Trabedersen) and vaccination techniques (Lucanix, Vigil). In addition, other approaches, such as antibodies targeting latent TGFβ complexes have been examined in preclinical studies [50]. All clinically approved Hh pathway inhibitors to date are Smo antagonists, such as vismodegib, sonidegib and glasdegib [31,32]. Disease relevant non-canonical TGFβ and Hh signaling, involving ligand-independent pathway activation through cross talk with other signaling pathways, often cannot be effectively targeted by these agents [13,14]. Oxysterol-based inhibitors, such as Oxy186 and Oxy210, display uniquely different mechanisms of action, i.e., inhibition of Hh signaling downstream of Smo and inhibition of TGFβ signaling independent of TGFβ receptor kinase inhibition, and may present a new opportunity for targeting both canonical and some of the disease relevant non-canonical TGFβ and Hh signaling. 

While the origins of the TGFβ and Hh signaling inhibitory properties await further study and elucidation, the inhibitory mechanism of Oxy210 is new and different from the known modalities of TGFβ and Hh inhibition currently available in the clinic or under clinical investigation. In future studies, we plan to examine the in vivo efficacy of Oxy210 in xenograft models of NSCLC and will continue with the assessment of its pharmacokinetic and safety profiles. 

## 5. Conclusions

Following our published report regarding the anti-Hh signaling effects of Oxy16 (Figure 1), a naturally occurring oxysterol, we screened numerous semi-synthetic analogues of Oxy16 in cell-based assays and selected Oxy210 as a lead compound based on: (1) the significantly enhanced biological activity of Oxy210 in inhibiting Hh as well as TGFβ signaling in vitro in NIH3T3 and human NSCLC cells relative to Oxy16, (2) ease of synthesis and scale up, and (3) favorable metabolic stability derived from in vitro human and mouse liver microsomal assays. Furthermore, oral dosing of Oxy210 in mice demonstrated a favorable absorption and PK profile and was well tolerated and apparently safe in mice with repeated once daily oral dosing at 50 mg/kg, 200 mg/kg and 400 mg/kg for 7 days. In summary, we have identified and characterized Oxy210 as a potential drug development candidate for NSCLC that displays a unique combination of TGFβ and Hh signaling inhibitory properties. Furthermore, our studies suggest that Hh inhibitory sterol molecules, such as Oxy16, Oxy186 and Oxy210, display different conformational preferences compared to Hh stimulatory sterol molecules, such as Oxy8 and Oxy133. The inhibitory mechanism of Oxy210 is new and different from the known modalities of TGFβ or Hh signaling inhibition currently available in the clinic or under clinical investigation: Oxy210 is not a TGFβ receptor kinase inhibitor, and, unlike Smoothened (Smo) antagonists, it inhibits Hh signaling downstream of Smo.

## Figures and Tables

**Table 1:** Metabolic Stability in human and mouse liver microsomes (HLM, MLM).

Compound ^1^	HLM, % Remaining after 60 min	HLM, Half-Life	MLM, % Remaining after 60 min	MLM, Half-Life
Oxy16	6%	15 min	5%	14 min
Oxy210	55%	69 min	61%	84 min

^1^ Metabolic stability testing was performed at Eurofins, Inc., according to methods described by Obach et al. [44].

## Supplementary Materials

Experimental write-up for the preparation of Oxy210, IC_50_ determinations for Figure 4b and Figure 7a–c, densitometry readings for Figure 11a are available online at https://www.mdpi.com/2073-4409/8/10/1297/s1.
