# Supplementary material for: Inhibition of Non-Small Cell Lung Cancer Cells by Oxy210, an Oxysterol-Derivative that Antagonizes TGFβ and Hedgehog Signaling
Source: Cells. 2019 Oct 22;8(10):1297. doi: 10.3390/cells8101297 (PMC6829886; doi:10.3390/cells8101297)
Supplement: Supplementary file 1 [file cells-08-01297-s001.zip › Experimental procedures for Oxy210 .pdf]

Synthesis of Oxy210:

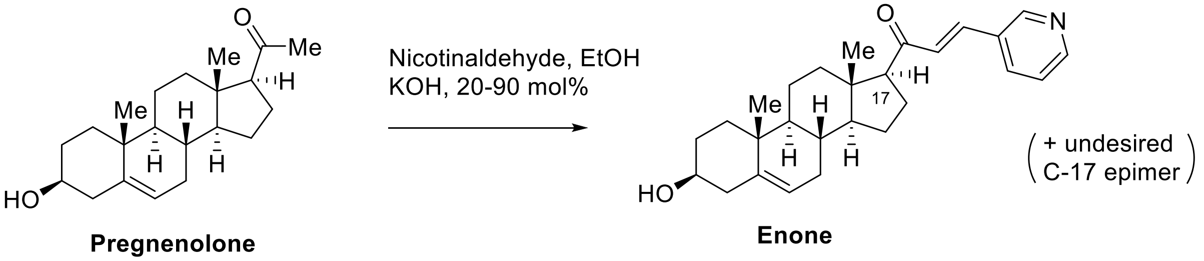

Step 1: Pregnenolone (5.0 g, 16 mmol) was suspended in ethanol (80 mL) at room temperature. A sub-stoichiometric amount* of potassium hydroxide solution (4M, 2 mL, 0.5 equivalents) was added to the reaction mixture followed by addition of nicotinaldehyde (1.78 mL, 19 mmol). The resulting mixture was stirred at room temperature for 24 hours. Upon completion of the reaction (TLC analysis), water (100 mL) was added to the reaction mixture to precipitate the product. The crude solid product was isolated using vacuum filtration, washed with water (2 x 50 mL) and then air dried. The crude product was used without further purification when free of undesired C-17 epimer (^{1}H-NMR analysis). Minor amounts of undesired C-17 epimer can be removed by hot filtration: the crude product was suspended in 1:1 hexane/ethyl acetate (25 mg/mL) and sonicated for several minutes. The undesired C-17 epimer was then removed through heating the mixture to reflux temperature for 10 mins followed by hot filtration of the desired product. There was obtained 6.68 g (67%) of enone product.

^{1}H NMR (400 MHz, $CDCl_3$) δ 8.75 (1H, d, J= 2 Hz), 8.57 (1H, dd, J= 5, 2Hz), 7.86-7.81 (1H, m), 7.50 (1H, d, J= 17Hz), 7.31 (1H, dd, J= 8, 4 Hz), 6.81 (1H, d, J= 17 Hz), 5.38-5.30 (1H, m), 3.56-3.40 (1H, m), 2.83 (1H, dd, J= 9, 9 Hz), 2.39-2.17 (3H, m), 2.06-1.95 (3H, m), 1.87-1.01 (13H, m), 0.98 (3H, s), 0.63 (3H, s); ^{13}C NMR (125 MHz, $CDCl_3$) δ 199.9, 150.3, 149.8, 140.9, 137.6, 134.6, 130.7, 128.5, 123.8, 121.3, 71.6, 62.3, 57.2, 50.0, 45.1, 42.3, 39.2, 37.3, 36.5, 32.0, 31.8, 31.6, 24.7, 22.7, 21.1, 19.4, 13.5.

* The use of excess potassium hydroxide favors the formations of the undesired C-17 epimer.

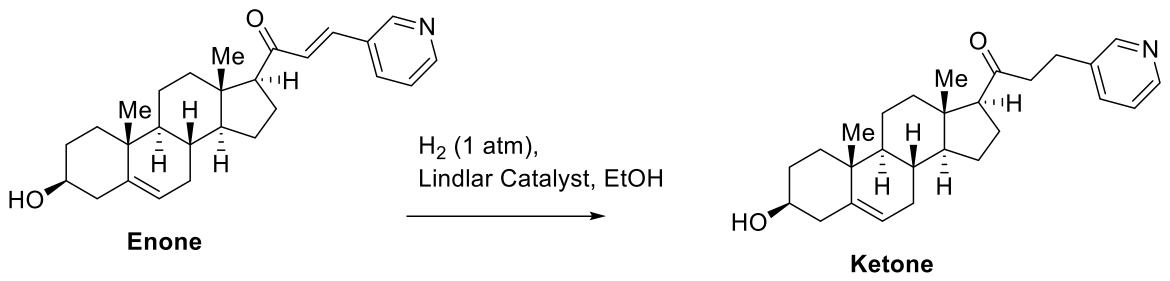

Step 2: The enone (4.0 g, 10 mmol) was suspended in ethanol (150 mL) at room temperature and Lindlar's catalyst (0.4 g) was added to the mixture. The atmosphere in the reaction flask was purged three times with hydrogen gas using a balloon. The reaction mixture was then stirred at room temperature under a hydrogen atmosphere. After 2 days, the mixture was filtered over celite and concentrated in vacuo to yield the crude ketone (4.0 g of recovery). The crude ketone product was dissolved in a minimal volume of hot ethyl acetate (heat gun) and allowed to crystallize overnight. The mother liquor was then removed from the crystalline solid by decantation. There was obtained 3.5 g of pure ketone product.

^{1}H NMR (400 MHz, $CDCl_3$) δ 8.42 (1H, d, J = 2Hz), 8.40 (1H, dd, J = 5,1 Hz), 7.52-7.47 (1H, m), 7.17 (1H, dd, J= 8, 5 Hz), 5.33-5.26 (1H, m), 3.54-3.44 (1H, m), 2.92-2.91 (2H, m), 2.72-2.64 (2H, m), 2.45, (1H, dd, J= 9, 9 Hz), 2.35-1.00 (17H, m), 0.96 (3H, s), 0.52 (3H, s); ^{13}C NMR (125 MHz, $CDCl_3$) δ 209.7, 149.7, 147.4, 140.8, 136.8, 136.2 123.3, 121.1, 71.4, 63.0, 56.9, 49.9, 45.3, 44.3, 42.2, 38.9, 37.2, 36.4, 31.8, 31.7, 31.5, 26.7, 24.4, 23.0, 21.0, 19.3, 13.3.

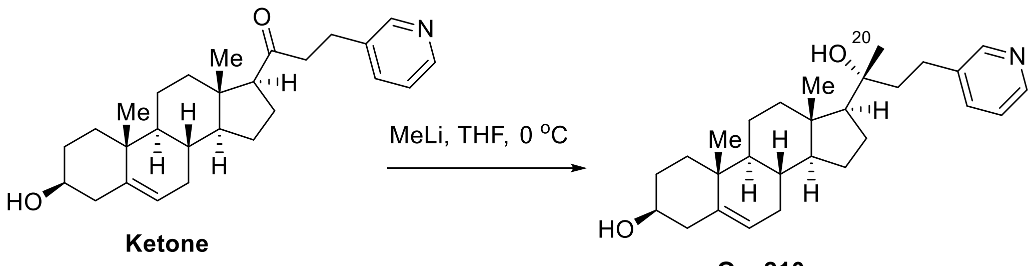

Step 3: The ketone (0.4 g, 1 mmol) was dissolved in dry tetrahydrofuran (5 mL) at room temperature and cooled to 0 °C under N_2-atmosphere. A solution of methyl lithium (1.4 M in ether, 7.2 mL, 10 mmol) was added dropwise to the reaction mixture at 0 °C. The reaction mixture was then stirred at 0 °C for 1 hour until the starting material was mostly consumed (TLC analysis). Then the reaction was carefully quenched with a small volume (~1 mL) of methanol and the mixture further diluted with saturated ammonium chloride solution (20 mL) and dichloromethane (20 mL). The layers were separated, and the aqueous layer extracted with dichloromethane (2 x 30 mL). The combined organic layers were dried over sodium sulfate, filtered and concentrated in vacuo. The crude mixture was purified via automated chromatography (ISCO) running an ethyl acetate/methanol gradient (0-10%) to yield pure Oxy210 product (0.17 g, 40%), recovered ketone starting material (0.09 g, 22%) and mixed fractions of starting material and product.

^{1}H NMR (400 MHz, $CDCl_3$) δ 8.45 (1H, d, J= 1Hz), 8.42 (1H, dd, J= 5, 2 Hz), 7.53-7.48 (1H, m), 7.23-7.18 (1H, m), 5.35-5.31 (1H, m), 3.56-3.45 (1H, m), 2.79-2.63 (2H, m), 2.33-2.17 (2H, m), 2.05 (1H, m), 2.01-1.26 (16 H, m), 1.23 (3H, s), 1.18-0.89 (3H, m), 0.98 (3H, s), 0.87 (3H, s); ^{13}C NMR (100 MHz, $CDCl_3$) δ 149.7, 147.1, 140.8, 138.1, 135.8, 128.6, 123.4, 121.4, 75.5, 71.6, 58.7, 56.9, 50.0, 44.1, 42.9, 42.3, 40.3, 37.2, 36.5, 31.7, 31.6, 31.3, 27.5, 26.7, 23.7, 23.2, 20.9, 19.3, 13.7.
